# A randomized controlled trial of a brain-computer interface based attention training program for ADHD

**DOI:** 10.1371/journal.pone.0216225

**Published:** 2019-05-21

**Authors:** Choon Guan Lim, Xue Wei Wendy Poh, Shuen Sheng Daniel Fung, Cuntai Guan, Dianne Bautista, Yin Bun Cheung, Haihong Zhang, Si Ning Yeo, Ranga Krishnan, Tih Shih Lee

**Affiliations:** 1 Department of Child and Adolescent Psychiatry, Institute of Mental Health, Singapore, Singapore; 2 School of Computer Science and Engineering, Nanyang Technological University, Singapore, Singapore; 3 Department of Biostatistics, Singapore Clinical Research Institute, Singapore, Singapore; 4 Center for Quantitative Medicine, Duke-National University of Singapore Medical School, Singapore, Singapore; 5 Center for Child Health Research, University of Tampere and Tampere University Hospital, Finland; 6 Neural & Biomedical Technology Department, Institute for Infocomm Research A*STAR, Singapore, Singapore; 7 Neuroscience and Behavioral Disorders Program, Duke-National University of Singapore Medical School, Singapore, Singapore; IRCCS E. Medea, ITALY

## Abstract

**Objective:**

The use of brain-computer interface in neurofeedback therapy for attention deficit hyperactivity disorder (ADHD) is a relatively new approach. We conducted a randomized controlled trial (RCT) to determine whether an 8-week brain computer interface (BCI)-based attention training program improved inattentive symptoms in children with ADHD compared to a waitlist-control group, and the effects of a subsequent 12-week lower-intensity training.

**Study design:**

We randomized 172 children aged 6–12 attending an outpatient child psychiatry clinic diagnosed with inattentive or combined subtypes of ADHD and not receiving concurrent pharmacotherapy or behavioral intervention to either the intervention or waitlist-control group. Intervention involved 3 sessions of BCI-based training for 8 weeks, followed by 3 training sessions per month over the subsequent 12 weeks. The waitlist-control group received similar 20-week intervention after a wait-time of 8 weeks.

**Results:**

The participants’ mean age was 8.6 years (SD = 1.51), with 147 males (85.5%) and 25 females (14.5%). Modified intention to treat analyzes conducted on 163 participants with at least one follow-up rating showed that at 8 weeks, clinician-rated inattentive symptoms on the ADHD-Rating Scale (ADHD-RS) was reduced by 3.5 (SD 3.97) in the intervention group compared to 1.9 (SD 4.42) in the waitlist-control group (between-group difference of 1.6; 95% CI 0.3 to 2.9 p = 0.0177). At the end of the full 20-week treatment, the mean reduction (pre-post BCI) of the pooled group was 3.2 (95% CI 2.4 to 4.1).

**Conclusion:**

The results suggest that the BCI-based attention training program can improve ADHD symptoms after a minimum of 24 sessions and maintenance training may sustain this improvement. This intervention may be an option for treating milder cases or as an adjunctive treatment.

## Introduction

Attention deficit hyperactivity disorder (ADHD) is a developmental disorder of childhood onset which can persist into adulthood[1] with negative academic and socio-occupational outcomes[2, 3]. Worldwide, including Singapore, ADHD is associated with significant burden[4, 5]. Current evidence-based treatment modalities include medication, psychosocial or behavioral treatment, or both[6, 7]. Long term data from studies such as the Multimodal Treatment of ADHD (MTA) study, however, did not provide a clear answer about which approach would give the best outcome in the long term[8, 9]. Neurofeedback therapy is a promising approach based on normalizing abnormal EEG patterns in children with ADHD, with trials demonstrating non-inferiority to medication and increased efficacy in combination with medication[10–14].

More recently, electroencephalogram (EEG)-based brain-computer interface (BCI) technology has been studied as well[15–18].

The BCI can quantify one’s attention level as measured by EEG waves, which we have harnessed to drive a series of training games (Cogoland). Our BCI system breaks down EEG waves into various frequency sub-bands covering the range from 4Hz to 36Hz (i.e. covering theta, alpha, beta 1, and beta 2 waves), which are further analyzed. We applied the machine learning method to derive a parametric model from the multi-band EEG signals, and then used this model to classify incoming EEG into attention or non-attention states, with a corresponding quantifiable score to indicate the subject’s attention level. Our first pilot trial involved the use of 3-channel EEG signals from the frontal (Fp1, Fp2) and parietal (Pz) positions. We successfully reduced to 2 frontal leads using a headband with dry EEG leads in our second pilot trial. Unlike traditional neurofeedback which teaches the child to suppress or enhance specific EEG waves based on prior assessment, our BCI system creates the individualized EEG pattern representing optimal attention based on the training activities (S1 File). Cogoland trains the individual to produce and maintain an optimal attentive state to proceed in the game, thereby motivating one to learn and sustain their attention to drive the game[19]. During the first 8 weeks of training, there will be a 10-minute academic task every alternate session, to help the individual generalize the learnt ability to regulate their concentration to everyday academic work.

Our earlier trials showed that we could detect improvement after 8 weeks of training, and that dropout rates became significant when the treatment period was longer. To compensate for sudden cessation of training, we included ‘maintenance’ training sessions (one session per month for 3 months) to examine if this was of any benefit. The entire BCI-based attention training program therefore would last 20 weeks. This randomized controlled trial (RCT) aimed to investigate the efficacy of this 20-week BCI-based attention training program. We expect most of the improvement to take place after the initial 8-week treatment, and hypothesize that the inattentive symptoms in the treatment group will be significantly reduced compared to untreated controls. Our secondary aim is to examine the effect of subsequent low-intensity maintenance training.

We had originally planned to used a sham-control design, which would greatly improve the quality of the study, but decided against it for several reasons. Most importantly, our experience with the local population is that most parents would decline to participate in clinical trials if there is a risk of receiving ‘placebo’ treatment. A sham-controlled trial will likely severely hamper recruitment or significantly increase drop-out rate. It is also dififcult to develop an adequate sham that the participants could not decipher. Additionally, it is arguable if it is ethical for participants to put in significant time and resource and yet only receive ‘sham’ treatment. If the ‘sham’ group were to then subsequently receive BCI intervention, the long duration of involvement would likely further increase drop-out. A sham-controlled trial design is therefore unlikely to succeed. As such, we decided on a waitlist-control design and ensured all participants would receive intervention at some point.

## Materials and methods

This study was supported by a grant from National Medical Research Council of Singapore [Grant Number CIRG11nov087 (to TSL)] and was approved by the ethics review board of the National Healthcare Group, Singapore (S1 Study Protocol). Written informed consent from parents and assent from children were obtained prior to study entry.

Clinical trial registration: https://www.clinicaltrials.gov; NCT01344044

### Trial design

We conducted a single-center, gender- and age-stratified (6 to 9 years and 10 to 12 years of age), outcome-assessor-blinded, waitlist-controlled, parallel-group study at the Child Guidance Clinic of the Institute of Mental Health in Singapore from January 2013 to June 2016. The intervention group received BCI-based attention training for 20 weeks and were followed up for a further 4 weeks. The control group served as untreated waitlist-controls for the first 8 weeks, after which they received BCI-based attention training for 20 weeks, and were similarly followed up for a further 4 weeks.

### Participants

**Fig 1:**
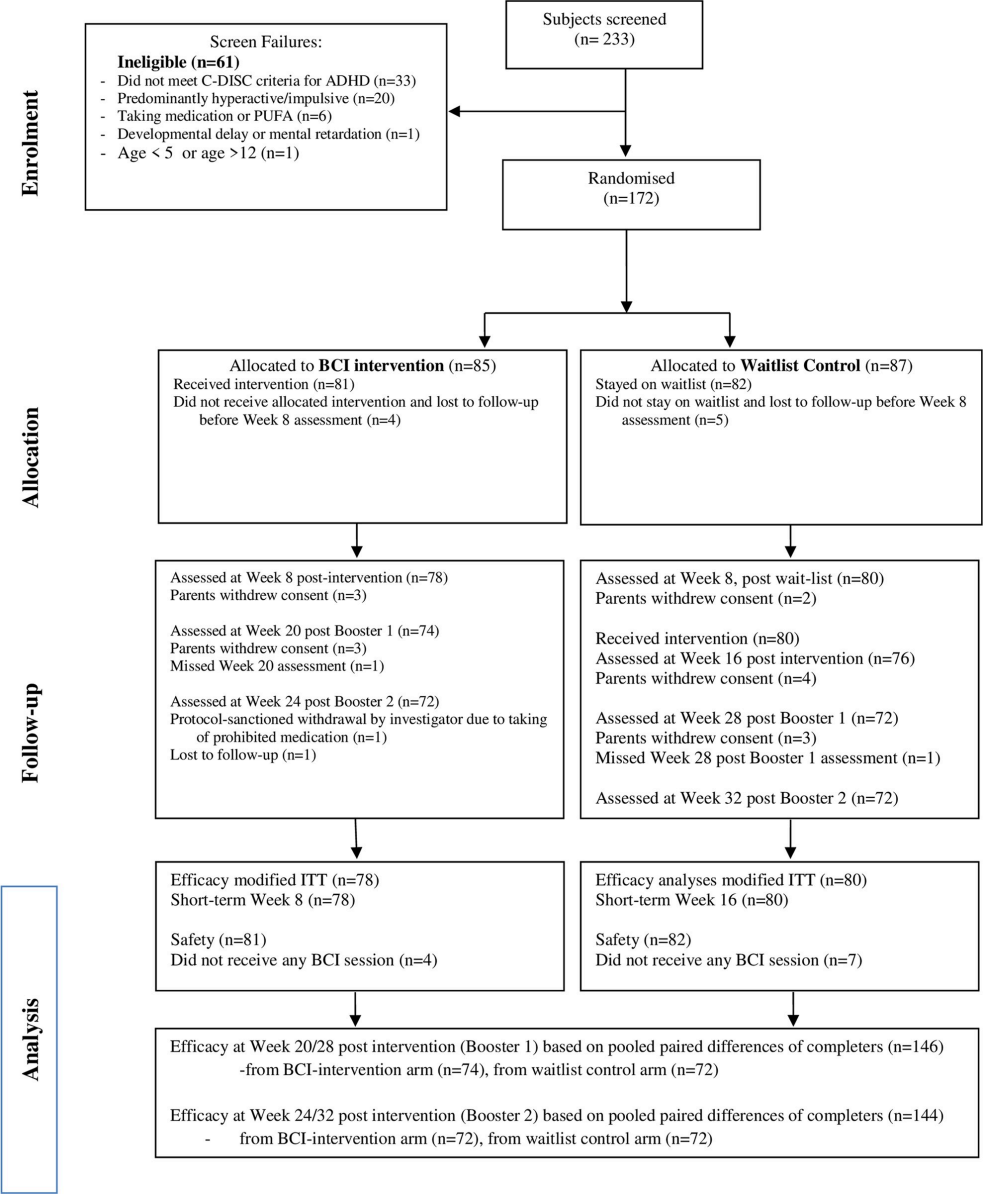
Participant flowchart (CONSORT flowchart).

We recruited children aged 6 to 12 years attending our outpatient psychiatric clinic who were diagnosed with ADHD based on the Diagnostic and Statistical Manual of Mental Disorders-Fourth Edition, text revised (DSM-IV TR). Those who had previously received pharmacotherapy or at least one month of fish oil supplementation had to undergo a washout period of at least 4 weeks. We excluded children with intellectual disability, epilepsy and severe sensori-neural deficits or co-existing psychiatric disorder which would interfere with their ability to complete the computer-based training activities. Parents completed the Computerized Diagnostic Interview Schedule for Children Version IV (CDISC-IV) and only those with significant inattentive symptoms (i.e. positive for a diagnosis of either inattentive or combined type of ADHD) were enrolled in the study[20]. Fig 1 (CONSORT flowchart) provides details about the participant flow throughout the course of the study.

### Intervention program

The BCI-based attention training game system consisted of a head-band with dry EEG electrode sensors that transmitted EEG readings to the computer through Bluetooth-enabled protocol (S1 File). We adopted the same 20-week training program as our second pilot study. The first 8 weeks (intensive phase) consisted of thrice-weekly sessions (24 sessions in total), following this, the next 12 weeks (maintenance phase) consisted of 4-weekly sessions (3 sessions in total).

Gantt schedule (S1 Fig) illustrates the study timeline and respective intervention period for both groups.

During the intensive phase, at the end of every alternate training session starting from the second session, each participant would complete twenty English and Mathematics questions and would be instructed to concentrate just like during their training. This task aims to help the child generalize the skill learnt to real life. The academic tasks would be administered during each session of the maintenance training.

Each participants would undergo the following tasks during the training sessions.

### Calibration

Prior to playing the game (Cogoland) at pre-BCI and upon completion of the program at post-BCI intervention, each participant underwent individual calibration using a colour Stroop task on the BCI-based game system. Critical EEG parameters during the correct attempts were analyzed and compared to the participants’ resting state, deriving an individualized EEG pattern representing the participant’s most attentive state.

### Cogoland

**Fig 2:**
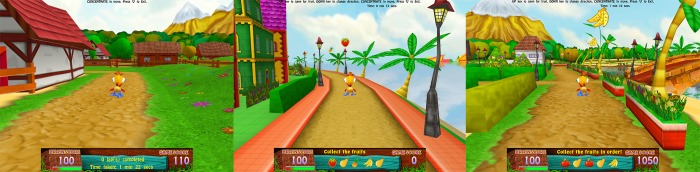
Cogoland game interface. There are three difficulty levels in Cogoland. The main goal of the basic level (left) is to drive the avatar around the island to complete as many laps as possible in ten minutes. The intermediate level (middle), participants are to drive the avatar around the island and collect fruits item presented at the bottom of the screen. At the advanced level (right), participants are to collect the fruits in order as presented on screen. Participants will use a specific key on the keyboard provided to make the avatar jump to collect the fruits. During each training session, participants will complete two 10-minute games, and a short break will be provided between the games.

The BCI-based intervention is a 3-D computerized graphic game developed with the intention to train attention (Fig 2). Participants will wear a BCI headset that detects their brainwaves using dry EEG electrodes during the training sessions. EEG data is transmitted to the computer via Bluetooth technology andanalyzed by an algorithm which drives the game interface, allowing participants to control the speed of their avatar with their attention level. The ‘higher’ the concentration level of the participant, the higher the speed of the avatar’s movement.

### Assessments

The following questionnaires were chosen to assess the treatment outcomes at various timepoints. With reference to the Gantt schedule (S1 Fig), the ‘intensive phase’ refers to the 8 weeks of intervention for both groups, ‘maintenance phase’ is represented by the subsequent 12 weeks of intervenition (Week 8 to 20 for intervention group and Week 16 to 28 for waitlist-control), and ‘long-term effect’ refers to the outcome at 4 weeks post-intervention (at Week 24 for intervention group, and Week 32 for wait-list control).

### ADHD rating scale (ADHD-RS)

The ADHD-RS is an 18-item rating scale used to rate the frequency of ADHD symptoms based on the DSM-IV TR[21]. The scores for the inattentive and hyperactive-impulsive symptoms add up to a maximum of 27: the higher the scores, the more severe the symptoms. One parent and one of two blinded clinicians on the study team completed the ADHD-RS at these time points:

- Weeks 0 and 8 for both groups
- Weeks 20 (end of maintenance phase) and 24 for the intervention group
- Weeks 16 (end of treatment phase), 28 (end of maintenance phase) and week 32 for the waitlist-control group.

The rating clinicians were blind to the treatment allocation and participants’ progress. They interviewed parents and obtained information from teachers when completing the scale.

### Child behavior checklist (CBCL)

The CBCL is a parent-rated questionnaire which yields information on a child’s behavioral/emotional problems. Scores are summarized for eight syndromes (Aggressive Behavior; Anxious/Depressed; Attention Problem; Rule Breaking Behavior; Social Problems; Somatic Complaints; Thought Problems; and Withdrawn/Depressed) and Internalizing, Externalizing, and Total Problems, as well as six DSM-oriented diagnoses scores including Affective Problems, Anxiety Problems, Attention Deficit/ Hyperactivity Problems, Oppositional Defiant Problems, Conduct Problems and Somatic Problems. T scores are also used to indicate whether the behavior falls within a clinical range[22].

### Pediatrics adverse events rating scale (PAERS)

The therapist checked with all participants before, during and at the end of each intervention if they had experienced any discomfort. Any adverse event identified was recorded and measured using the PAERS[23].

### Outcomes

The primary outcome was the short-term effect in the clinician-rated inattention symptoms as assessed by the Inattention score of the ADHD-RS. Additionally, 3 aspects of efficacy were evaluated as indicated in S1 Fig. The short-term efficacy compared change scores from Week 0 to Week 8 between both groups. The maintanence effect examined pooled change scores from Week 0 to Week 20 in the BCI-intervention group and Week 8 to Week 28 in the waitlist-control group, while the long-term effect examined pooled change scores from Week 0 to Week 24 in the BCI-intervention group and Week 8 to Week 32 in the waitlist-control group.

The secondary efficacy outcomes consisted of short-term changes in the parent-rated ADHD-RS Inattention Score, the CBCL ADHD Problems (one of six DSM diagnoses), Internalizing and Externalizing t-scores. We compared change scores from group-specific set of initial and endpoint values to assess for the outcome at the end of the 20-week intervention and at 4 weeks post-intervention (end of trial).

Safety outcomes consisted of the number and proportion of patients reporting treatment-emergent adverse events as recorded in the PAERS as well as the type, (worst) severity and frequency of each adverse event.

### Sample size

To detect a moderate effect size of 0.5 on the clinician-rated ADHD-RS Inattention score (i.e. the difference in mean change from Week 0 to 8 between BCI-intervention and waitlist-control groups), with a two-sided 5% significance level and a power of 80%, a sample size of 80 subjects per group was necessary, allowing for up to 20% losses to follow-up[24, 25].

### Randomization

The randomization sequence was created using Statistical Analysis Software (SAS) 9.3 (New Cary, North Carolina, USA) by the Singapore Clinical Research Institute (SCRI), a third party research organization without clinical involvement in the trial. The randomization sequence was stratified by gender and age (6 to 9 years and 10–12 years) with a 1:1 allocation using random permuted block sizes of 4 and 6. Upon the patients’ signing of informed consent and assent, authorized study personnel used a password-protected web-based system that implemented the randomization sequence. The system automatically assigns the eligible patients to the treatment groups upon entering patient’s initials and details of stratification factors.

### Blinding

Whereas patients, their parents and study investigators were aware of the treatment allocation, clinicians who conducted outcome assessments and the teachers who provided feedback were kept blinded to the treatment allocation. Patients and their parents were regularly advised not to inform the clinicians and teachers of the group the patients were allocated.

### Data analysis

To address the primary hypothesis of evaluating a short-term BCI treatment effect, we used the two-sample t-test to compare the mean change in the clinician-rated ADHD-RS Inattention Score from Week 0 to Week 8 between the BCI-intervention and waitlist-control groups (S1 Dataset). The difference in means between the two groups and its 95% confidence interval are reported. We used the same procedure to analyze secondary efficacy endpoints, namely parent-rated ADHD-RS Inattention score and t-scores in the CBCL Inattention, Internalizing and Externalizing domains. Analyzes were performed on a modified intent-to-treat population, that is all randomized patients who had non-missing Week 8 assessments, irrespective of eligibility status or compliance with protocol, and analyzing them under the group they were randomized to.

We did not impute missing Week 8 assessments, but conducted sensitivity analyzes using a mixed model repeated measures (MMRM) approach using all available data to verify robustness of the results[26]. In the model specification, we included the fixed categorical effects of gender (M or F), age stratum (6–9 years or 10–12 years), treatment allocation (BCI-intervention or waitlist-control), time (Week 0, 8, 20, 24 for BCI-intervention and 0, 8, 16, 28 and 32 for waitlist-control) and the treatment-by-visit interaction as well as the fixed continuous covariate of Week 0 score. We specified a group-specific Toeplitz covariance structure to model the within-patient effects and the Kenward-Roger approximation to estimate denominator degrees of freedom. The primary treatment comparison was the contrast in least squares means between the two groups at the Week 8 visit.

To assess the effect of the 20-week intervention program, we pooled paired data from both groups. The paired data consisted of change in efficacy endpoints as rated by clinicians and parents at week 20 from week 0 in the BCI-intervention group and changes at week 28 from week 8 in the waitlist-control group. We subtracted post-BCI from pre-BCI scores, and a positive change score indicated an improvement. We then summarized the change scores using means and standard deviations and tested for significance using the paired t-test. We quantified uncertainty by reporting 95% confidence intervals.

We followed up on all subjects 4 weeks after treatment completion and analyzed these results using change in efficacy endpoints at Week 24 from week 0 in the BCI-intervention group and changes at Week 32 from Week 8 in the waitlist-control group. We used the same MMRM model described earlier to visualize the longitudinal changes in clinician-rated ADHD-RS Inattention scores in both groups.

To assess safety, we included all participants who received at least one BCI session, pooling data from both groups. We obtained the number and proportion of participants reporting treatment-emergent adverse events with an onset date on or after date of first use of the BCI device. We also obtained the type, (worst) severity and frequency of treatment-emergent adverse effects. We used counts and proportions to assess safety of the BCI device.

Where applicable, all tests of hypotheses were two-tailed and performed at a 5% level of significance. Statistical analyzes were implemented using SAS 9.4 (SAS Institute, Cary North Carolina, USA).

## Results

### Study participation

A total of 172 participants were recruited in the study, including 147 males (85.5%) and 25 females (14.5%). The trial commenced in January 2013, and ended in June 2016 after recruitment target of 172 participants were met. Table 1 provides a summary of their demographic and clinical characteristics. Their mean age was 8.6 (SD = 1.51). The groups appear comparable in most of their baseline characteristics. With respect to CDISC-type of ADHD, there were 15% more participants of the combined subtype in the waitlist-control group than the BCI-intervention group.

**Table 1:** Baseline socio-demographic and clinical characteristics.

	BCI-Intervention (n = 81)	Waitlist control (n = 82)	Total (n = 163)
**Age, Mean (SD)**	8.7 (1.37)	8.6 (1.69)	8.6 (1.54)
**Gender**			
Male	69 (85.2)	69 (84.1)	138 (84.7)
Female	12 (14.8)	13 (15.9)	25 (15.3)
**Ethnicity**			
Chinese	72 (88.9)	74 (90.2)	146 (89.6)
Malay	3 (3.7)	3 (3.7)	6 (3.7)
Indian	4 (4.9)	2 (2.4)	6 (3.7)
Others	2 (2.5)	3 (3.7)	5 (3.1)
**Children Global Assessment Scale**			
n	66	60	126
Mean (SD)	63.2 (7.39)	61.3 (10.57)	62.3 (9.06)
Median (IQR)	64.0 (10.0)	61.5 (9.5)	62.0 (10.0)
**CDISC-type of ADHD**			
Combined	41 (50.6)	54 (65.9)	95 (58.3)
Inattention	40 (49.4)	28 (34.1)	68 (41.7)
**ADHD-RS Inattention score, Mean (SD)**	18.9 (4.25)	18.6 (4.38)	18.7 (4.31)

### Primary outcome

The description and analysis of clinican-rated ADHD-RS scores are presented in Table 2. There was a mean change (improvement) of 3.5 (SD 3.87) points on the inattentive symptom score at week 8 for the BCI-intervention group on the clinician-rated ADHD-RS, compared to a corresponding mean change of 1.9 (SD 4.42) points for the untreated controls. The mean difference (MD) was 1.6 (95% CI 0.3 to 2.9, p = 0.0177).

**Table 2:** Primary efficacy (m-ITT) analysis: Clinician-rated ADHD-RS, Inattention score.

Time	BCI-Intervention (n = 81)		Waitlist control (n = 82)		Effect size	
					T-test	MMRM
	Mean	SD	Mean	SD	MD (95% CI)	MD (95% CI)
**Short-term efficacy (post 8-week training)**						
Week 0	18.9	4.25	18.6	4.38	1.6 (0.3 to 2.9) p = 0.0177^a^	1.5^b^ (0.2 to 2.3) p = 0.0218
Week 8	15.5	4.48	16.7	5.14		
Change*	3.5	3.87	1.9	4.42		
**Post-intervention (20-week training)**						
Week 0/8^c^	18.9	4.25	16.7	5.14	2.4^e^ (1.6 to 3.2) p<0.0001^f^	2.4 (1.6 to 3.1) p<0.0001
Week 20/28^c^	15.6^d^	5.26^d^	15.6^d^	5.12^d^		
Change*	3.3	5.55	1.4	3.94		
**4-week post-intervention**						
Week 0/8^c^	18.9	4.25	16.7	5.14	3.3^e^ (2.5 to 4.2) p<0.0001^f^	3.2 (2.5 to 3.9) p<0.0001
Week 24/32^c^	14.3^d^	5.68^d^	14.8^d^	5.09^d^		
Change*	4.7	5.94	2.0	4.26		

* Positive change scores indicate improvement from initial time-point

^a^ two-sample independent t-test, n = 158 (size of modified intent-to-treat population)

^b^ difference between least squares (LS) means estimated from mixed model repeated measures (MMRM) approach, n = 163

^c^ Change calculated from Week 0 in BCI-Intervention and Week 8 in Waitlist control

^d^ BCI sample sizes at Week 8, 20 and 24 are 78, 74 and 73 respectively; Waitlist control sample sizes at Week 8, 28 and 32 are 80, 72 and 72 respectively.

^e^ Mean change score from pooled BCI-Intervention and Waitlist control groups

^f^ One-sample t-test to test null hypothesis that mean is equal to zero

### Secondary efficacy outcome analyzes

The assessment of changes at the end of the 20-week intervention and at 4 weeks post-intervention refers to the mean of paired differences (pre-post BCI scores) in the pooled BCI-intervention and waitlist-control groups. Based on the clinician-rated ADHD-RS Inattention scores (see Table 2), the maintenance phase was associated with a mean change (improvement) of 2.4 points (95% CI 1.6 to 3.2) after completing the 20-week intervention program. The improvement was maintained 4 weeks post-intervention, as indicated by mean change from start of intervention to the end of trial (i.e., 4 weeks after completing intervention), 3.3 points (95% CI 2.5 to 4.2). Other secondary outcomes which were collected, the clinician-rated Children’s Global Assessment Scale (CGAS), Clinical Global Impression Scale (CGI-S) and Clinical Global Improvement Scale (CGI-I) have been summarized in S2 File and in S1 Table.

Parent-reported ADHD-RS inattentive symptom outcomes were consistent with the clinician-rated results in all three efficacy aspects (Table 3). At Week 8, participants randomized to the BCI intervention and waitlist-control groups both improved by 4.0 (SD 4.8) and 1.8 (4.21) points respectively. The difference in mean improvement between groups was 2.2 (95% CI 0.8 to 3.6, p = 0.0024). Pooling both groups, the mean improvement from pre-BCI to post-BCI training was 3.1 points (95% CI 2.2 to 3.9, p<0.0001) and the long-term efficacy from pre-BCI to the end of the trial was 3.8 points (95% CI: 2.8 to 4.7, p<0.0001). The magnitude of benefit due to, or associated with the BCI treatment effect are larger in the parent-reported compared to clinician-rated ADHD-RS.

**Table 3:** Secondary efficacy analysis: Parent-rated ADHD-RS, and CBCL.

Outcome	Treatment (n = 81)		Control (n = 82)		Effect size
**Parent-rated ADHD-RS**	Mean	SD	Mean	SD	Mean Difference (95% CI); p-value
Week 0	18.9	4.84	18.6	4.24	
Δ* at Week 8	4.0	4.80	1.8	4.21	2.2 (0.8 to 3.6); p = 0.0024^a^
Δ* at Week 20/28	4.1	5.59	2.1	4.49	3.1^b^ (2.2 to 3.9); p<0.0001^c^
Δ* at Week 24/32	5.3	6.17	2.2	4.66	3.8^b^ (2.8 to 4.7); p<0.0001^c^
**Parent-rated CBCL: Internalizing**	Mean	SD	Mean	SD	Mean Difference (95% CI); p-value
Week 0	61.2	10.1	60.9	10.59	
Δ* at Week 8	5.5	7.58	2.1	7.19	3.4 (1.0 to 5.7); p = 0.005 ^a^
Δ* at Week 20/28	6.5	8.76	2.1	9.36	4.3^b^ (2.8 to 5.9); p<0.001 ^c^
Δ* at Week 24/32	7.1	8.76	3.9	8.45	5.5^b^ (4.1 to 6.9); p<0.001 ^c^
**Parent-rated CBCL: Externalizing**	Mean	SD	Mean	SD	Mean Difference (95% CI); p-value
Week 0	62.5	9.45	64.6	9.19	
Δ* at Week 8	3.3	6.54	2.5	6.51	0.8 (-1.2 to 2.9); p = 0.417 ^a^
Δ* at Week 20/28	3.7	8.20	1.7	7.16	2.7^b^ (1.4 to 4.0); p<0.001 ^c^
Δ* at Week 24/32	4.7	8.70	1.9	7.40	3.3^b^ (1.9 to 4.6); p<0.001 ^c^

* Δ = change score; Δ > 0 indicates improvement, Δ = 0 no change and Δ < 0 deterioration; in Waitlist control group, Δ is calculated from Week 8 when the child starts intervention, for changes at end of intervention (week 28) and end of trial (week 32).

^a^ Mean difference, independent two-sample t-test

^b^ Mean of change scores from pooled BCI-treatment and Waitlist control groups

^c^ One-sample t-test result to test null hypothesis that mean score is zero

Parent-rated CBCL externalizing and and internalizing problem score found change (reduction) in the internalizing problem score of 5.5 (SD 7.58) in the BCI-treatment group to be significantly greater than the reduction of 2.1 (SD 7.19) seen in the waitlist-control group (MD 3.4, 95% CI 1.0 to 5.7, p = 0.005) at week 8 compared to week 0. However, the change in externalizing problem scores was not statistically significant between groups (MD 0.8, 95% CI -1.2 to 2.9, p = 0.417) (see Table 3).

### Sensitivity analyzes on clinician-rated ADHD-RS inattention score based on MMRM

**Fig 3:**
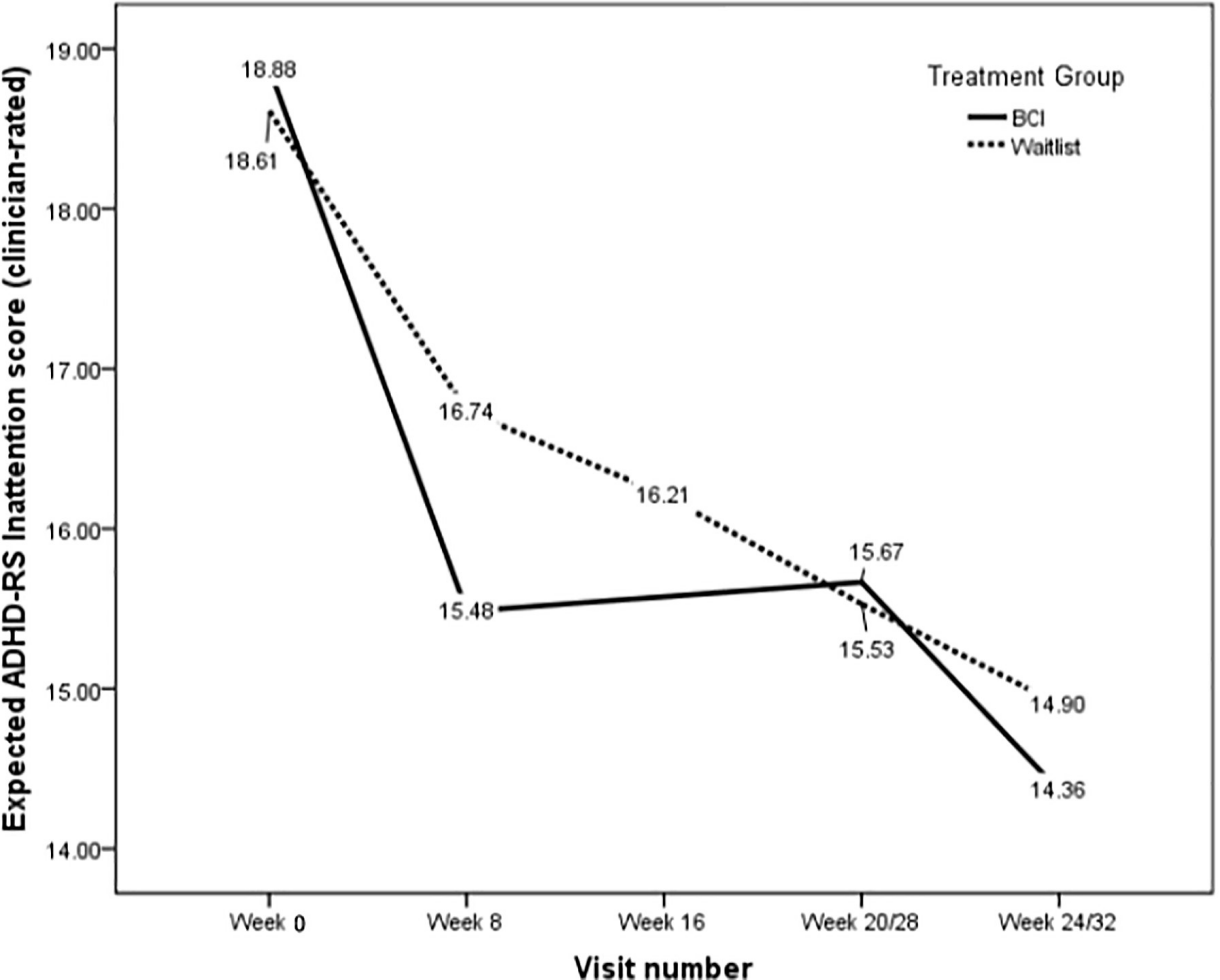
Analysis and results of clinician-rated ADHD-RS inattention score.

Key results of sensitivity analyzes based on MMRM approach, which is robust to data missing at random, is shown in Fig 3. Details of difference estimates and their 95% CIs from the MMRM are shown in the last column of Table 2. The data pattern is broadly consistent with the main analysis presented above. At the short-term, improvement was significantly greater in subjects randomized to the intervention group (3.4 points, 95% CI: 2.5 to 4.4) compared with subjects in the waitlist-control (1.9 points, 95% CI: 0.4 to 3.3), or a mean difference of 1.5 points (95% CI: 0.2 to 2.3, p = 0.0218). At the end of maintenance training (Week 20/28), the improvement from pre-BCI (week 0 in BCI-treatment and week 8 in waitlist-control) was sustained in both groups. The paired pre-post BCI mean difference was 3.2 (95% CI: 1.9 to 4.6) and 1.2 (95% CI: 0.2 to 2.4) points in theintervention and waitlist-control groups respectively. The pooled mean change was 2.4 (95% CI 1.6 to 3.1) (see Table 2). At Week 4 after the end of maintenance training (Week 24/32), the improvement from pre-BCI (week 0 in intervention and week 8 in waitlist-control) persisted in both groups, that is, neither reversion nor deterioration in inattentive scores were seen. The paired pre-post BCI mean difference was 4.5 (95% CI: 3.1 to 5.6) and 1.8 (95% CI: 0.7 to 3.3) points in the BCI-intervention and waitlist-control groups respectively, or a pooled mean change of 3.2 (95% CI 2.5 to 3.9). The inattentive symptom profiles in both groups were consistent with the hypothesized trend.

### Adverse events

A total of 11 (6.4%) children reported at least one adverse event and headache was the commonest complaint, followed by dizziness (S2 Table). Only 1 participant reported 2 different adverse events–headache and trouble paying attention/concentrating–on one occasion. None of these adverse events required medical treatment or was rated to be severe. In most cases, the participants were able to carry on with the intervention session after they were given a short break.

## Discussion

There is a plethora of research exploring alternative treatment modalities such as supplementation, dietary restriction and complementary treatment, although none appear to have therapeutic efficacy matching medication[27]. In search for an alternative therapy for ADHD which can be safer and yet effective, researchers have looked into other executive deficits commonly identified in children with ADHD[28].

The results showed that the BCI-based attention training program could improve the inattentive symptoms of ADHD after 8 weeks of 20 to 24 intervention sessions, when compared to untreated controls. The short-term efficacy effect size was small (about 0.4 SD) when rated by blinded clinicians but moderate according to parents’ ratings. Among untreated controls, there was a small overall improvement in the inattentive symptoms after an 8-week waitlist period, based on both clinicians’ and parents’ ratings. This could be the result of being involved in a trial (‘halo effect’) or parents could have implemented behavioral strategies to manage their ADHD symptoms. Parents today are often well-read and may have received behavioral management strategies from their previous treating psychiatrists. The subsequent monthly maintenance training did not appear to further improve the inattentive symptoms, but may have some value in sustaining the gains made.

During the trial, there were few adverse events and none of them required further medical attention. The commonest adverse events were headache and giddiness. These were likely to be related to the discomfort from the headset, having to sustain their attention for a period of time and the effect of staring at the computer screen. Compared to our pilot trials which we used an EEG cap and a different brand of headset respectively, the incidence of adverse events were lower in this trial. This could in part be due to the relative comfort of the headset which has an outer cushion lining. The headset can also be adjusted to fit the child’s head size although it cannot be too loose as there needs to be close contact between the electrodes and the skin for the EEG waves to be accurately picked up.

When we designed this novel intervention program, we drew inference from previous studies on cognitive training including neurofeedback therapy, on the number of training sessions needed. Neurofeedback therapy is based on the hypothesis that correcting abnormalities on the EEG related to cortical slowing, such as increased relative theta power and increased theta/beta power ratios, would result in improvement of clinical symptoms[29]. The literature suggested that the minimum number of sessions was 20, and could go up to 80 sessions to train the individual to modify their EEG waves. We were limited to using the shortest training schedule in our trial as most parents preferred to participate during their children’s school holidays which spanned about 8 weeks. Our pilot trials showed that 24 sessions over an 8-week period could be effective and the dropout rate was not excessive. With the available knowledge, we therefore defined treatment completers as those who completed a minimum of 20 sessions. There is a possibility that further increasing the number of training sessions can improve the inattentive symptoms more and this is a possible area for future studies to explore.

Studies across different populations have suggested that children and adolescents with ADHD can experience significant anxiety and mood symptoms which can further add to their impairment[30, 31]. Following treatment with the BCI-based attention training program, the children in our study showed improvement in their internalizing symptoms. There was however no statistically significant improvement in the attention problem subscale although we did not power our study to detect this. This improvement in internalizing symptoms could be secondary to the reduction in their ADHD symptoms. As their ADHD symptoms improved, they would become more likely to experience less impairment and negative experiences, which would in turn reduce their anxiety and negative mood. However, as these were parent-reported, it could also be possible that parents assessed their child to experience less internalizing problems upon noticing reduction in ADHD symptoms and negative behaviors. Alleviating these internalizing symptoms is important during treatment as co-existing anxiety symptoms has been associated with poorer outcome in children with ADHD as compared to their peers[32].

It would have been ideal if we could get information from the teachers who would have been ideal blinded assessors, but our pilot trial experience showed that it was difficult to get an adequate response rate from teachers. Hence we decided to utilize blinded clinician ratings instead for our primary outcome. Consistent with other studies showing that the male to female ratio tends to be more skewed in clinical than community samples, almost 90% of our participants were males. This could limit the generalisability of the trial results to the population. Also, parents who participated could be more likely to be motivated and there would be possibility of response bias.

The advantage of our BCI-based attention training program over most other cognitive training programs utilizing EEG information is that it is relatively simple to use. As the equipment needed is only a headset and a computer, there is potential for this intervention to be developed into a home-based treatment. This can make treatment more convenient and accessible. Its relative safety is also likely to make it more acceptable to parents compared to pharmacotherapy with the associated medication-related side effects.

## Conclusions

This RCT demonstrated improvement in inattentive symptoms among children with ADHD with our BCI-based attention training program. This intervention is also safe, engaging and well-tolerated. It can potentially be used to treat milder ADHD symptoms or as an adjunctive therapy. Future trials can investigate the effects of different intensities of training. This approach can be developed into a home-based treatment option and offer increased convenience and accessibility.

## Supporting information

S1 CONSORT Checklist (PDF)

S1 Study Protocol Study protocol 2009/00395. (PDF)

S1 File BCI neurofeedback system. (PDF)

S2 File Secondary efficacy analysis: Clinician-rated CGAS, CGI-S and CGI-I. (PDF)

S1 Fig Gantt schedule illustrating the respective intervention and assessments conducted for both groups, and the timepoints for efficacy analyses. The primary outcome (i.e. short term efficacy) was the clinician-rated ADHD-RS at Week 8. Secondary outcomes (i.e. maintenance efficacy and short efficacy) included clinician-rated ADHD-RS, parent-rated ADHD-RS and CBCL at the respective timepoints: for BCI-intervention group, these were Week 20 (maintenance efficacy) and Week 24 (long term efficacy); for waitlist control group, these were Week 28 (maintenance efficacy) and Week 32 (long term efficacy).
(TIF)

S1 Dataset (XLSX)

S1 Table Secondary efficacy analysis: Clinician-rated CGAS, CGI-S and CGI-I. (PDF)

S2 Table Summary of adverse events for all subjects who underwent at least one BCI session. (PDF)

## References

[1] Faraone SV, Biederman J, Mick E. The age-dependent decline of attention deficit hyperactivity disorder: a meta-analysis of follow-up studies. Psychol Med. 2006;36(2):159–65. 10.1017/S003329170500471X . 16420712

[2] Dalsgaard S, Mortensen PB, Frydenberg M, Thomsen PH. Long-term criminal outcome of children with attention deficit hyperactivity disorder. Crim Behav Ment Health. 2013;23(2):86–98. 10.1002/cbm.1860 . 23576439

[3] Mohr-Jensen C, Steinhausen HC. A meta-analysis and systematic review of the risks associated with childhood attention-deficit hyperactivity disorder on long-term outcome of arrests, convictions, and incarcerations. Clin Psychol Rev. 2016;48:32–42. 10.1016/j.cpr.2016.05.002 . 27390061

[4] Health EDCDMo. Singapore Burden of Disease Study 2010 2014.

[5] Phua HP, Chua AV, Ma S, Heng D, Chew SK. Singapore's burden of disease and injury 2004. Singapore Med J. 2009;50(5):468–78. . 19495514

[6] Chan E, Fogler JM, Hammerness PG. Treatment of Attention-Deficit/Hyperactivity Disorder in Adolescents: A Systematic Review. JAMA. 2016;315(18):1997–2008. 10.1001/jama.2016.5453 . 27163988

[7] Fung DS, Lim CG, Wong JC, Ng KH, Cheok CC, Kiing JS, et al Academy of Medicine-Ministry of Health clinical practice guidelines: attention deficit hyperactivity disorder. Singapore Med J. 2014;55(8):411–4; quiz 5. 10.11622/smedj.2014098 25189301 PMC4294089

[8] Molina BS, Hinshaw SP, Swanson JM, Arnold LE, Vitiello B, Jensen PS, et al The MTA at 8 years: prospective follow-up of children treated for combined-type ADHD in a multisite study. J Am Acad Child Adolesc Psychiatry. 2009;48(5):484–500. 10.1097/CHI.0b013e31819c23d0 19318991 PMC3063150

[9] Parker J, Wales G, Chalhoub N, Harpin V. The long-term outcomes of interventions for the management of attention-deficit hyperactivity disorder in children and adolescents: a systematic review of randomized controlled trials. Psychol Res Behav Manag. 2013;6:87–99. 10.2147/PRBM.S49114 24082796 PMC3785407

[10] Goode AP, Coeytaux RR, Maslow GR, Davis N, Hill S, Namdari B, et al Nonpharmacologic Treatments for Attention-Deficit/Hyperactivity Disorder: A Systematic Review. Pediatrics. 2018;141(6). 10.1542/peds.2018-0094 . 29848556

[11] Jiang Y, Abiri R, Zhao X. Tuning Up the Old Brain with New Tricks: Attention Training via Neurofeedback. Frontiers in Aging Neuroscience 2017;9(52):9. 28348527 10.3389/fnagi.2017.00052 PMC5346575

[12] Zuberer A, Brandeis D, Drechsler R. Are treatment effects of neurofeedback training in children with ADHD related to the successful regulation of brain activity? A review on the learning of regulation of brain activity and a contribution to the discussion on specificity. Front Hum Neurosci. 2015;9:135 10.3389/fnhum.2015.00135 25870550 PMC4376076

[13] Baumeister S, Wolf I, Hohmann S, Holz N, Boecker-Schlier R, Banaschewski T, et al The impact of successful learning of self-regulation on reward processing in children with ADHD using fMRI. ADHD Attention Deficit and Hyperactivity Disorders. 2018 10.1007/s12402-018-0269-6 30225805

[14] Pakdaman F, Irani F, Tajikzadeh F, Jabalkandi SA. The efficacy of Ritalin in ADHD children under neurofeedback training. Neurol Sci. 2018 10.1007/s10072-018-3539-3 . 30187306

[15] Sterman MB. Physiological origins and functional correlates of EEG rhythmic activities: Implications for self-regulation. Biofeedback and Self-Regulation. 1996;21(1):3–33. 8833314 10.1007/BF02214147

[16] Arns M, Batail JM, Bioulac S, Congedo M, Daudet C, Drapier D, et al Neurofeedback: One of today's techniques in psychiatry? Encephale. 2017;43(2):135–45. 10.1016/j.encep.2016.11.003 . 28041692

[17] Angelakis E, Hatzis A, Panourias IG, Sakas DE. Brain-computer interface: a reciprocal self-regulated neuromodulation. Acta Neurochir Suppl. 2007;97(Pt 2):555–9. . 17691347 10.1007/978-3-211-33081-4_64

[18] Carelli L, Solca F, Faini A, Meriggi P, Sangalli D, Cipresso P, et al Brain-Computer Interface for Clinical Purposes: Cognitive Assessment and Rehabilitation. Biomed Res Int. 2017;2017:1695290 10.1155/2017/1695290 28913349 PMC5587953

[19] McDermott AF, Rose M, Norris T, Gordon E. A Novel Feed-Forward Modeling System Leads to Sustained Improvements in Attention and Academic Performance. J Atten Disord. 2016 10.1177/1087054715623044 . 26823382

[20] Shaffer D, Fisher P, Lucas CP, Dulcan MK, Schwab-Stone ME. NIMH Diagnostic Interview Schedule for Children Version IV (NIMH DISC-IV): description, differences from previous versions, and reliability of some common diagnoses. J Am Acad Child Adolesc Psychiatry. 2000;39(1):28–38. 10.1097/00004583-200001000-00014 . 10638065

[21] DuPaul GJ, Reid R, Anastopoulos AD, Lambert MC, Watkins MW, Power TJ. Parent and teacher ratings of attention-deficit/hyperactivity disorder symptoms: Factor structure and normative data. Psychol Assess. 2016;28(2):214–25. 10.1037/pas0000166 . 26011476

[22] Achenbach TM, Rescorla LA. Manual for the ASEBA school-age forms & profiles Burlington, VT: University of Vermont, Research Center for Children, Youth, & Families2001.

[23] March J, Karayal O, Chrisman A, editors. CAPTN: The Pediatric Adverse Event Rating Scale. Annual Meeting of the American Academy of Child and Adolescent Psychiatry 2007 23–28 October 2007; Boston.

[24] Cohen J. A Power Primer. Psychological Bullein. 1992;112(1):155–9. 10.1037//0033-2909.112.1.155 19565683

[25] Cohen J. Statistical power analysis for the behavioral sciences 2 ed. Abingdon, United Kingdom: Routledge; 1988.

[26] Fairclough DL. Design and analysis of quality of life studies in clinical trials United States of America: A CRC Press Company; 2002.

[27] Bloch MH, Qawasmi A. Omega-3 fatty acid supplementation for the treatment of children with attention-deficit/hyperactivity disorder symptomatology: systematic review and meta-analysis. J Am Acad Child Adolesc Psychiatry. 2011;50(10):991–1000. 10.1016/j.jaac.2011.06.008 21961774 PMC3625948

[28] Evans SW, Owens JS, Bunford N. Evidence-based psychosocial treatments for children and adolescents with attention-deficit/hyperactivity disorder. Journal of Clinical Child & Adolescent Psychology. 2014;43(4):527–51. 10.1080/15374416.2013.850700 PMC4025987 24245813

[29] Baumeister S, Wolf I, Holz N, Boecker-Schlier R, Adamo N, Holtmann M, et al Neurofeedback training effects on inhibitory brain activation in ADHD: A matter of learning? Neuroscience. 2016 10.1016/j.neuroscience.2016.09.025 . 27659116

[30] Jensen PS, Hinshaw SP, Kraemer HC, Lenora N, Newcorn JH, Abikoff HB, et al ADHD comorbidity findings from the MTA study: comparing comorbid subgroups. J Am Acad Child Adolesc Psychiatry. 2001;40(2):147–58. 10.1097/00004583-200102000-00009 . 11211363

[31] Xia W, Shen L, Zhang J. Comorbid anxiety and depression in school-aged children with attention deficit hyperactivity disorder (ADHD) and selfreported symptoms of ADHD, anxiety, and depression among parents of school-aged children with and without ADHD. Shanghai Arch Psychiatry. 2015;27(6):356–67. 10.11919/j.issn.1002-0829.215115 27199527 PMC4858507

[32] Sciberras E, Lycett K, Efron D, Mensah F, Gerner B, Hiscock H. Anxiety in children with attention-deficit/hyperactivity disorder. Pediatrics. 2014;133(5):801–8. 10.1542/peds.2013-3686 . 24753534

