# Supplementary material for: A randomized controlled trial of a brain-computer interface based attention training program for ADHD
Source: PLoS One. 2019 May 21;14(5):e0216225. doi: 10.1371/journal.pone.0216225 (PMC6528992; doi:10.1371/journal.pone.0216225)
Supplement: S1 Study Protocol — (PDF) [file pone.0216225.s002.pdf]

STUDY PROTOCOL

PROTOCOL TITLE:

A Randomised Controlled Trial of a Brain-Computer Interface Based Intervention for the Treatment of ADHD

PROTOCOL NUMBER: 2009/00395

PROTOCOL VERSION: 5.0

PROTOCOL DATE: 20 August 2015

PRINCIPAL INVESTIGATOR:

Dr Lim Choon Guan
Consultant and Deputy Chief
Department of Child and Adolescent Psychiatry
Institute of Mental Health
Buangkok Green Medical Park, 10 Buangkok View, Singapore 539747

MAIN STUDY SITE:

Institute of Mental Health
Buangkok Green Medical Park, 10 Buangkok View, Singapore 539747

Co-Investigators:

Associate Professor Lee TihShih
Duke NUS Graduate Medical School
8 College Road, Singapore 169857

Associate Professor Daniel Fung
Adjunct Associate Professor
Senior Consultant
Chairman Medical Board

Institute of Mental Health
Buangkok Green Medical Park, 10 Buangkok View, Singapore 539747

Professor Cheung Yin Bun
Chief Scientific Officer
Singapore Clinical Research Institute
31 Biopolis Way, Nanos #02-01
Singapore 138669

Dr. Guan Cuntai
Programme Manager
Intelligent Systems for Personalized and Connected Healthcare
A*STAR Institute for Infocomm Research
1 Fusionopolis Way, Connexis (South Tower) #21-01, Singapore 138632

Collaborator:

Dr Zhang Hai Hong

Intelligent Systems for Personalized and Connected Healthcare
A*STAR Institute for Infocomm Research
1 Fusionopolis Way, Connexis (South Tower) #21-01, Singapore 138632

Protocol Synopsis

Study Objective

This study aims to examine the efficacy of a brain-computer interface (BCI) system for the treatment of inattentive symptoms of Attention Deficit Hyperactivity Disorder (ADHD). Our hypothesis is that children with ADHD receiving treatment with the BCI-based training will improve to a greater extent than the control group.

Study Population

This study will enroll ADHD children aged 6 to 12 referred from Child Guidance Clinic (CGC)

Eligibility Criteria

The child should satisfy the following inclusion criteria:

1. Between the age of 6 and 12 years old inclusive.
2. Satisfy the following criteria for the diagnosis of ADHD:
   - Meets criteria for ADHD, combined or inattentive subtypes based on the Computerized Diagnostic Interview Schedule for Children (C-DISC).

They should not have any of the following exclusion criteria:

1. Present or history of medical treatment with stimulant medication and Atomoxetine in the past 1 month.
2. Present or regular intake of polyunsaturated fatty acids supplement intake (e.g. Omega-3 oil, flax seed oil, cod liver oil) for more than 2 weeks in the past 3 months.
3. Present or history of traditional Chinese medicine (TCM) prescribed for treatment of attention problems in the past 1 month.
4. Co-morbid severe psychiatric condition or known sensory-neural deficit e.g. complete blindness or deafness.
5. History of epileptic seizures.
6. Known to have developmental delay (i.e. IQ 70 and below).
7. Predominantly hyperactive/impulsive subtype of ADHD.

Research Related Activities

Subjects will complete structured interviews, questionnaires and undergo assessments in the clinic.

Intervention for the subjects will include the following:

1. BCI training protocol.

Assessments for the participants involved will include the following:

1. Children: KBIT-2, Conner's Continuous Performance Test, 2nd Edition (CPT-II).
2. Parents: C-DISC, Attention Deficit Hyperactive Disorder- Rating Scale (ADHD-RS) and Child Behaviour Checklist (CBCL).

3. Clinicians: ADHD-RS, Clinical Global Assessment Scale (C-GAS), Clinical Global Impression Severity Scale (CGI-S), Clinical Global Impression Improvement Scale (CGI-I) and Pediatric Adverse Event Rating Scale (PAERS).

Table of Contents

Protocol Synopsis 3
Study Objective 3
Study Population 3
Eligibility Criteria 3
Research Related Activities 3
1. Background and Rationale 7
1.1 Rationale and justification for the Study 7
2. Hypothesis and Objectives 8
2.1.1 Primary Objective 8
2.1.2 Primary Endpoint 8
2.2.1 Secondary Objectives 8
2.3.1 Secondary Endpoints 9
2.4.1 Safety Endpoints 9
2.5.1 Exploratory analysis 9
3. Study Population 9
3.1 Inclusion Criteria 9
3.2 Exclusion Criteria 10
3.3 Withdrawal Criteria 10
4. Trial Schedule 10
5. Study Design 11
5.1 Intervention Group 12
5.2 Control Group 12
6. Methods and Assessments 13
6.1 Selection of Subjects 13
6.2 Enrolment from Clinic .... **Error! Bookmark not defined.**
6.3 Briefing session for all potential subjects 13
6.4 Randomisation 14
6.5 Blinding 14
6.6 Study Visits and Procedure 15

7. Trial Device 17
8. Treatment of Subjects 17
8.1 Specific Restrictions 17
8.2 Concomitant Medications 17
8.3 Unblinding 18
9. Safety Measurements 18
9.1 Definitions 18
9.1.1 Adverse Events 18
9.1.2 Unanticipated Problems Involving Risk to Subjects or Others 18
9.1.3 Serious Adverse Events 18
9.2 Collection, Recording and Reporting 19
9.2.1 AE 19
9.2.2 UPIRTSO 19
9.3 Safety Monitoring Plan 19
9.4 Complaint Handling 19
9.5 Criteria for Stopping the Research 19
10. Data Analysis 20
10.1 Data Quality Assurance 20
11. Sample Size and Statistical Method 20
11.1 Determination of Sample size 20
11.2 Statistical Analysis Plans 20
12. Ethical Considerations 21
12.1 Informed Consent by the Parent and Informed Assent by the Child 21
12.2 Confidentiality of Data and Patient Records 22
13. Publication Policy 22
14. Retention of Trial Documents 22
15. Insurance 23
16. References 23
17. Appendices 24
17.1 List of Abbreviations 24

1. Background and Rationale

ADHD is the most common psychiatric condition among children and adolescents seen at the CGC in Singapore much like the rest of the world. ADHD is diagnosed clinically and is characterised by symptoms of inattention and/or hyperactivity/ impulsivity, with onset in childhood (1). Community studies have found the prevalence of ADHD to be between 1.7% and 16% (2). Recent studies demonstrated that up to half of the children with ADHD continue to have symptoms in adulthood (1, 3). Male-female ratios range from 9:1 to 6:1 in clinical samples but are about 3:1 in community-based population studies (4). ADHD is associated with academic underachievement, work difficulty, social rejection, driving accidents, smoking, alcohol and drug abuse, and poor self esteem. In view of the prevalence, persistence and long term impairment, ADHD is an important public health problem. About 500 new cases of ADHD are referred to CGC annually for treatment, and the number is still increasing.

Management of ADHD includes pharmacological and behavioural management. Pharmacological treatment includes stimulant and non-stimulant medications (1, 5). Methylphenidate is the only stimulant medication available in Singapore. Parents are often concerned about its side effects including poor appetite, physical growth retardation and cardiovascular effects. Being a potential drug of abuse, methylphenidate is also avoided in patients who may be likely to abuse the medication. On the other hand, Atomoxetine, a relatively new non-stimulant medication, is expensive with significant side effects. Atomoxetine carries a 'black box label' warning of potential increased suicidal thinking associated with its use. Behavioural management, though important, has been shown to be less superior to pharmacological treatment (6). Follow-up results of the NIMH Collaborative Multisite Multimodal Treatment Study of Children With Attention-Deficit/Hyperactivity Disorder (MTA) showed that despite initial symptom improvement during treatment, the superiority of medication was lost by 3 years, and the 8-year follow up showed that children with combined-type ADHD exhibit significant impairment in adolescence (7).

BCI is a direct communication pathway between a human brain and an external device. It is a technology that enables people to interact with computers through their thoughts. Electroencephalogram (EEG) is the best studied non-invasive interface facilitating such communication, mainly due to its fine temporal resolution, ease of use, portability and low set-up cost. It presents the user with real-time feedback on brainwave activity, as measured by electrodes on the scalp, typically in the form of a video display, sound or vibration (8). In children with ADHD, reduced levels of the higher-frequency brain waves are especially noticeable in the prefrontal cortex, an area involved in attention control. They also have an increase in lower-frequency waves, especially theta waves from 4 to 7.5 Hz. Neuro-feedback, a form of biofeedback programme (9, 10, 11), has been used in a limited fashion but is not well studied.

1.1 Rationale and justification for the Study

We have developed a series of BCI-based training activities incorporating our patented attention detection technology into interactive games for the treatment of ADHD. As the BCI extracts a quantifiable attention level from EEG, users can employ their attention to play these games directly.

Therefore, there is no need for the users to follow any prompt or output on the screen; in other words, this works in an 'asynchronous' mode. With such a mode, user can start to play the game straightaway, without any learning. At the same time, since the user can control a game without any feedback (auditory or visual) there is a unique feed-forward mechanism as well. To help the user progress through the series of activities of increasing difficulty, players will first need to master a simple concentration task before moving on to the next task according to the treatment protocol. Most therapy involves at least a total of 20 sessions (11); as such, our intervention protocol includes 20 sessions followed by 3 one-monthly 'booster training'. The treatment protocol also involves a homework task for every alternate session, during which the child will be 'trained' to use their newly-acquired concentration skill during everyday tasks. Our intervention and treatment protocol requires little training for the therapist, and the equipment required is simple and portable.

A pilot non-randomized trial to test the treatment protocol and its efficacy was conducted on 10 children with ADHD. Another 10 children with ADHD were recruited as controls. The 20–session intervention program was well accepted by the parents and children. The ADHD scales administered by parents and teachers both showed good test-retest reliability (>0.9). We have found satisfactory level of compliance to the intervention protocols.

Therapy using non-invasive BCI system-based games may represent one alternative means to treat children with ADHD symptoms. If demonstrated to be efficacious, parents are likely to prefer this as an alternative to current treatment modalities of medications (with potentially serious side effects) and behavior management (parents generally find this difficult and challenging). Children themselves are also more likely to enjoy the treatment and hence, comply better.

In the longer term, this approach and system may have potential for attention and cognitive enhancement for patients with cognitive impairment or early dementia.

2. Hypothesis and Objectives

2.1.1 Primary Objective

This study aims to examine the efficacy of a brain-computer interface system for the treatment of inattentive symptoms of Attention Deficit Hyperactivity Disorder (ADHD). The hypothesis is that children with ADHD receiving treatment with the BCI-based training will improve to a greater extent than the control group.

2.1.2 Primary Endpoint

The primary outcome of this study will be the change in the inattentive score on the ADHD Rating Scale as rated by the clinicians from week 1 to week 8.

2.2.1 Secondary Objectives

- To investigate the sustainability of the effect of a brain computer interface system for the treatment of inattentive symptoms of Attention Deficit Hyperactivity Disorder (ADHD).

- To investigate the effect of a brain computer interface on daily functioning.
- To investigate if there is any safety concern related to the use of BCI.

2.2.2 Secondary Endpoints

1) The change in the inattentive score of the ADHD RS rated by parent from week 1 to week 8.
2) The change in the inattentive score of the ADHD RS rated by clinician and parent from week 1 to week 8, week 1 to week 20 and week 1 to week 24 for the intervention group and week 8 to week 16, week 8 to week 28 and week 8 to week 32 for the control group.
3) The change in parent rated CBCL inattention score from week 1 to week 8.
4) The change in parent rated CBCL inattention score from week 1 to week 8, week 1 to week 20 and week 1 to week 24 for the intervention group and week 8 to week 16, week 8 to week 28 and week 8 to week 32 for the control group.
5) The change in clinician rated CGAS and CGI scores from week 1 to week 8.
6) The change in clinician rated CGAS and CGI scores from week 1 to week 8, week 1 to week 20 and week 1 to week 28 (intervention group) and week 8 to week 16, week 8 to week 28 and week 8 to week 32 (control group).

2.3.1 Safety Endpoints

Adverse events and serious adverse events.

2.4.1 Exploratory analysis

Investigate the level of agreement in symptom severity among reports from parents and clinicians, as well as performance on CPT.

3. Study Population

This study will enroll 172 children age 6 to 12 years with ADHD.

3.1 Inclusion Criteria

A subject will be eligible for inclusion in the study if ALL of the following criteria apply:

1. The child should be between the age of 6 and 12 years old inclusive
2. The child should satisfy the following criteria for the diagnosis of ADHD:
   - Meets criteria for ADHD, combined or inattentive subtypes based on the Computerized

     Diagnostic Interview Schedule for children (C-DISC)

3.2 Exclusion Criteria

A subject will not be eligible for inclusion in the study if ANY of the following criteria apply:
1. Present or history of medical treatment with stimulant medication and Atomoxetine in the past one month.
2. Present or regular intake of polyunsaturated fatty acids supplement intake (e.g. Omega-3 oil, flax seed oil, cod liver oil) for more than 2 weeks in the past 3 months.
3. Present or history of traditional Chinese medicine (TCM) prescribed for treatment of attention problems in the past 1 month.
4. Co-morbid severe psychiatric condition or known sensory-neural deficit e.g. complete blindness or deafness (such that they cannot play computer games).
5. History of epileptic seizures.
6. Known to have developmental delay (i.e. IQ 70 and below).
7. Predominantly hyperactive/impulsive subtype of ADHD (i.e. no predominant inattentive symptoms).

3.3 Withdrawal Criteria

Subjects may be withdrawn from the intervention according to the discretion of the Principal Investigator if:
1. ADHD-RS raw score increases by 25% as rated by clinicians, and/or
2. CGI-S scores are between 5 and 7 inclusive.
3. Receive any medication (e.g. methylphenidate, atomoxetine, tricyclic anti-depressant, and/or polyunsaturated fatty acids (PUFA) supplement (e.g. Omega-3 oil, flax seed oil, cod liver oil), and/or traditional Chinese medication (TCM) which can improve ADHD symptoms during the trial period. The parent/child will be asked about this each time they turn up at the clinic.

All recruited subjects are free to withdraw consent and discontinue participation without penalty and without providing reasons. However data collection will continue after withdrawal from intervention unless the subject or parents/legal guardians decide to withdraw from the study totally (this will be explicitly asked again upon withdrawal).

Participants can still choose to continue or discontinue with the remaining assessments at Week 1, 8, 16 (control), 20 (intervention), 24 (intervention), 28 (control), and 32 (control) despite having BCI training sessions being ceased.

Subjects will be considered as ‘drop-outs’ if they:
1. Become lost to follow-up or discontinues intervention.

4. Trial Schedule

During Pre-Screening, the parent/legal guardian will complete the structured interview, CDISC.

Once the diagnosis of ADHD (inattentive/combined subtype) has been made, the parent/legal guardian will complete the following questionnaires during the baseline stage (Week 1):

a. ADHD Rating Scale

b. Child Behavior Checklist

If the child has ever been placed in the school's Learning Support Program and/or is currently failing a subject, he/she will be screened using the Kaufman Brief Intelligence Test.

The assessment schedule is summarized in Table 1 section 6.6.

Once the diagnosis of ADHD has been made, the following assessments will be completed by different informants:

CLINICIAN:

1. ADHD Rating Scale (ADHD- RS)

2. Clinical Global Assessment Scale (CGAS)

3. Clinical Global Impression - Severity Scale (CGI-S)

4. Clinical Global Impression – Improvement Scale (CGI-I)

5. Pediatric Adverse Event Rating Scale (PAERS) from week 8 onwards as there will be nothing to report at the first session.

PARENT:

1. ADHD Rating Scale (ADHD-RS)
2. Child Behavior Checklist (CBCL)
3. CDISC

CHILD:

1. Conners' Continuous Performance Test (CPT-II)

The blinded clinicians, parents and children will complete their assessments at weeks 1, 8, 16 (control), 20 (intervention), 24 (intervention), 28 (control) and 32 (control). The children and their parents will come down to the clinic for these scheduled assessments. The blinded clinician will contact the child's school teacher(s) and obtain feedback about the child's behavior in school. This will be made known to parents during the briefing session held prior to enrolment.

5. Study Design

Our hypothesis is that children with ADHD receiving treatment with the BCI-based training will improve to a greater extent than the control group. This will be organized as a prospective single blind randomized clinical trial (clinicians blinded to subjects treatment arm), with the comparator arm being a

wait-list control. All subjects will eventually receive the BCI intervention but subjects in the control arm will not receive it until week 9.

5.1 Intervention Group

86 subjects in the intervention group will take part in a total of 24 sessions spread over an 8-week period, followed by one-monthly booster sessions for another 3 months and a final follow-up review 1 month after the completion of the training session. If sessions are missed during the 8-weeks period due to unforeseen circumstances (e.g. sickness, travel plans), arrangements will be made for participants to attend up to 5 BCI-based therapy sessions per week. All participants will have to complete a minimum of 20 sessions within the 8-weeks period for treatment efficacy.

Each subject will first need to master a simple concentration task before moving on to play a series of training tasks. Each task employs the BCI system, and is controlled by the child's concentration. As the child attends to activities on a computer screen, their EEG waves will be recorded simultaneously via the EEG sensors through Bluetooth technology.

In addition, as part of the treatment protocol, they will be tasked to do a set of MCQ on the computer. The task will consist of both Math and English questions and will be matched to their grade level. During the second session, subjects will be administered MCQs at their appropriate grade level.

Administering of MCQs will be done at alternate intervention sessions over the 8-week period and during each of the 3 monthly 'booster' sessions. Their EEG activity will be recorded during these tasks as well. Subjects will be advised to concentrate during the task just as they did during the BCI intervention. This will train the child to generalize their 'concentration skills' learnt during the BCI training to their academic tasks.

The children in this group will be followed up by their child psychiatrist at the following weeks: Week 1, 8, and 20. At the end of this study phase, the clinician will discuss with the parents about their child's condition and a progress report will be provided. If parents are keen, they will be referred to CGC or REACH for further follow up and treatment.

5.2 Control Group

86 subjects in the control group will not receive BCI training during the first 8 weeks of their study participation; they will act as controls. At week 9, subjects in this group will go through the BCI training similar to the intervention group. If sessions are missed during the 8-weeks period due to unforeseen circumstances (e.g. sickness, travel plans), arrangements will be made for participants to attend up to 5 BCI-based therapy sessions per week. All participants will have to complete a minimum of 20 sessions within the 8-weeks period for treatment efficacy.

They will take part in a total of 24 sessions spread over an 8-week period, followed by one-monthly booster sessions for another 3 months. Subjects will be tasked to do a set of MCQs on the computer. The task will consist of both Math and English questions and will be matched to their grade level. The procedure for MCQ administration will be exactly the same as the intervention group. Administering of MCQs will be done at the end of alternate intervention sessions over the 8-week period and during each

of the 3 monthly 'booster' sessions. Their EEG activity will be recorded during these tasks as well. Their participation in the research study will last 8 months. The subjects in the control group will complete the exact same questionnaires at the same time-points as the intervention group.

All children in this group will be followed up by their child psychiatrist at the following weeks: Week 1, 8, 16, 28 and 32.

6. Methods and Assessments

6.1 Selection of Subjects

This study will enroll 172 children aged between 6 to 12 years with ADHD from CGC.

6.2 Enrolment from Clinic

First contact with the subjects will be as follows:

The treating doctor will inform parents of potential subjects. An information letter will be provided to the parents of potential subjects to invite them to learn more about the study. After parents have indicated interest in knowing more about the BCI study, the protocol administrator will contact parents of potential subjects to arrange a clinic visit for a briefing session where the study will be explained to them in greater detail..

Potential subjects who are not enrolled with the clinic will be advised to register as a clinic patient first before recruitment into the study.

6.3 Briefing session for all potential subjects

During briefing sessions, the research team will provide an overview of ADHD and a demonstration of the BCI study will be conducted for parents to view. It will be highlighted that medication and psychosocial treatment represent current evidence-based treatment for this disorder. The BCI study will be presented as an alternative form of treatment that is being investigated in this study and parents will have ample time and opportunity to choose the treatment of preference. At the end of each briefing, parents will be provided with participant information sheets to take home for further consideration of participation.

After the briefings, parents will be contacted within one to two weeks to give all subjects sufficient time to contemplate and ask any queries pertaining to the study. If the parents indicate interest, a session will then be arranged for informed consent to be taken. Informed consent will be obtained from the parents, and assent from the subjects, prior to any form of assessment or intervention as part of the study. The blinded clinician will contact the child's school teacher(s) and obtain feedback about the child's behavior in school at various time points. This will be made known to parents during the briefing session. All trial-related procedures will only take place after informed consent has been obtained. A baseline assessment will be done to determine the child's eligibility for the study.

In the event that the child is eligible but the parents/legal guardians choose not to participate in the study, the parents/legal guardians will be offered an option to have the child be followed-up at the CGC.

Participation is strictly voluntary and at any point if the subject wishes to drop-out, he/she will not be restricted or punished for it.

6.4 Randomisation

This is a randomised, single-blind study. When the informed consent and assent forms are signed and the eligibility criteria are confirmed, the subject will be randomized to intervention or control group at a 1:1 allocation ratio, giving approximately 86 subjects in each group. Balanced treatment assignments will be achieved using permuted block randomisation, stratified by gender and age, across one study site. The stratification factor for age will be categorized into two groups, aged 6-9 years old and aged 10-12 years old. Block size will be determined by the study statistician and will not be made known to the clinical investigators or site personnel. The randomization procedure, done after obtaining consent, assent and CDISC results at Week 0, will assign a unique “subject number” to each child.

Implementation of randomisation will be carried out via a web-based program at SCRI. Authorized study centre personnel will randomise the patient via a password-protected internet web site. The randomisation system will then determine the treatment arm and provide the subject number to be used for the patient. The site monitor/CRA will be informed immediately in the event that the web randomization is not successful. Back-up randomisation envelopes will be provided to the site in case of internet failure.

6.5 Blinding

Clinicians conducting the study assessments will be blinded to the study arm. Teachers who will be providing feedback to the clinicians will be blinded as well. Study Investigators, study personnel (including the SCRI study team), parents and subjects are not blinded in this study. Parents and subjects will be advised regularly not to inform the clinicians or teachers to which group the subjects are assigned.

6.6 Study Visits and Procedure

Table 1: Study Schedule [Phase 2]

* Note: Group 1 = Intervention group
Group 2 = Control group

		Pre-Study Screening	Baseline	Treatment G1 / Control G2	Boosters G1 / Treatment G2		Boosters G2	Follow-Up	
**Week**		NIL	Week 1	Week 8	Week 16	Week 20	Week 28	Week 24	Week 32
**Duration of Visit**		2 hours	1 hour	75 min	30 min	60 min	60 min	30 min	30 min
Informed Consent	Both groups	XX							
Computerised Diagnostic Interview Schedule for Children [C-DISC] (Parent)	Group 1	XX							
	Group 2								
Conner's Continuous Performance Test II [CPT-II] (Child)	Group 1		XX	XX		X		X	
	Group 2				X		X		X
Kaufman Brief Intelligent Test [KBIT-2] (Child) [Selected]	Group 1								
	Group 2	XX							
Randomisation	Both groups	XX							
Parent Questionnaire									
Child Behaviour Checklist [CBCL] (113 items)	Group 1		XX	XX		X		X	
	Group 2				X		X		X
ADHD Rating Scale	Group 1		XX	XX		X		X	
	Group 2				X		X		X
Clinician Questionnaire									
Clinical Global Assessment Scale	Group 1		XX	XX		X		X	
	Group 2				X		X		X
Clinical Global Impression - Severity Scale	Group 1		XX	XX		X		X	
	Group 2				X		X		X
Clinical Global Impression - Improvement Scale	Group 1		XX	XX		X		X	
	Group 2				X		X		X
ADHD Rating Scale	Group 1		XX	XX		X		X	
	Group 2				X		X		X
Pediatric Adverse Event Rating Scale (PAERS)	Group 1			X		X		X	
	Group 2				X		X		X

NB: All assessment visits will be completed accordingly to the assessment week within the window period of +/- 5 working days.

Figure 1: Study Visit and Procedure for Both Groups

Appendix A

Schedule 1: Gantt schedule of Phase 2 overall study design and timelines for intervention & control groups.

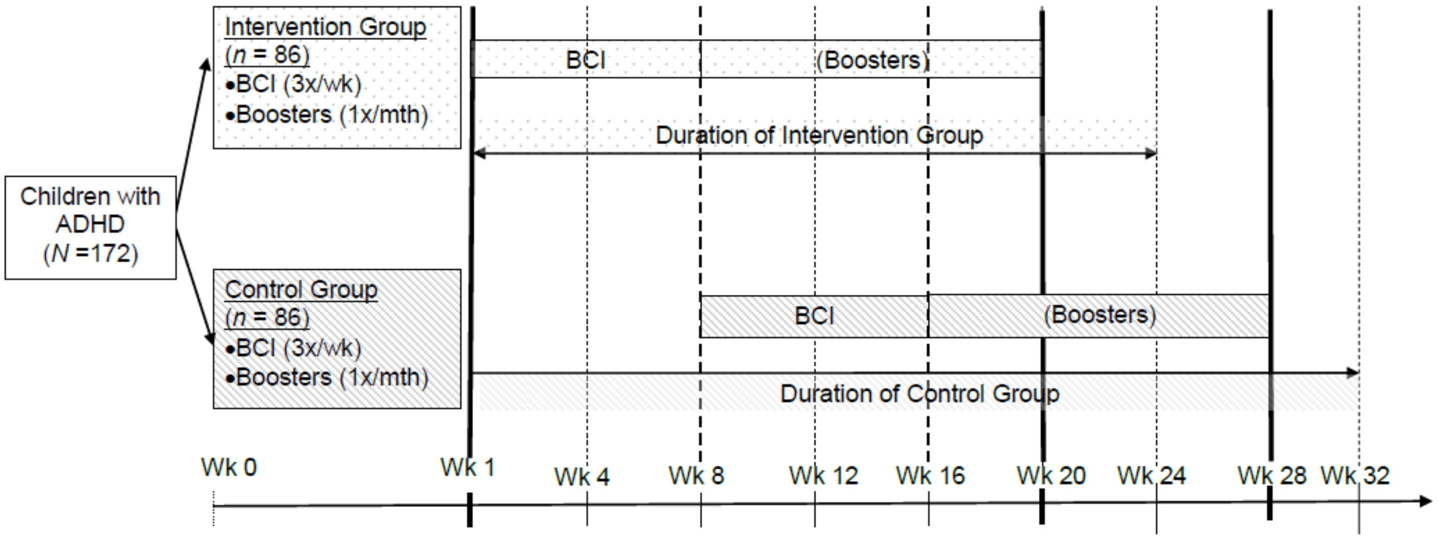

Assessments for both intervention and control group

Week 0	Week 1 (+/- 5 work days)	Week 8 (+/- 5 work days)	Week 16 (+/- 5 work days)	Week 20 (Intervention) /Week 28 (Control) (+/- 5 work days)	Week 24 (Intervention) /Week 32 (Control) (+/- 5 work days)
**Pre-screening**	**Baseline**	**Post-Tx** (Intervention) **Pre-Tx** (control)	**Post-Tx** (control)	**Post-booster**	**Follow-up**
**Parents:** C-DISC	**Parents**: ADHD-RS / CBCL	**Parents**: ADHD-RS / CBCL	**Parents**: ADHD-RS / CBCL	**Parents**: ADHD-RS / CBCL	**Parents**: ADHD-RS / CBCL
**Child:** K-BIT2 (selected)	**Clinician**: ADHD-RS C-GAS / CGI	**Clinician**: ADHD-RS C-GAS / CGI	**Clinician**: ADHD-RS C-GAS / CGI	**Clinician**: ADHD-RS C-GAS / CGI	**Clinician**: ADHD-RS C-GAS / CGI
**Randomisation**	**Child**: CPT	**Child**: CPT	**Child**: CPT	**Child**: CPT	**Child**: CPT

BCI Design and Schedule (Phase 2) DSRB A/09/395 Version 7.0 dated 01 April 2015 Page 1 of 2

7. Trial Device

The intervention is a non-invasive BCI system-based games that is unlikely to cause any direct harm and may improve ADHD symptoms.

While the subject play the training game and attempt the MCQ tasks, EEG waves will be recorded through EEG sensors that are connected through Bluetooth technology. The subject wears the headband with EEG sensors placed on the subject's forehead and a clip is attached to the ear lobe for grounding purposes.

8. Treatment of Subjects

8.1 Specific Restrictions

1. There are no dietary restrictions.

2. There is no limitation in the physical activity of the subject.

3. Intake of methylphenidate, atomoxetine and tricyclic anti-depressant are to be avoided 1 month before and during the study since these medications can improve ADHD symptoms. The parent/legal guardian and/or the subject will be asked about this during each study visit. If there is a need for these medications to be prescribed to the subject then the subject will be withdrawn from the study.

4. Intake of polyunsaturated fatty acids (PUFA)supplement intake (e.g. Omega-3 oil, flax seed oil, cod liver oil) which can improve ADHD symptoms are also to be avoided 3 months before and during the study. These supplements are receiving attention as a complementary approach to managing ADHD. While not a primary empirical based treatment and efficacy outcome varied, the study team will exclude subjects who are taking these supplements in the past 3 months, if they have been taking them regularly for a period longer than 2 weeks, to ensure that any improvement in inattentive symptoms is not due to the supplementation.

5. Intake of traditional Chinese medication (TCM) treatment is also to be avoided 1 month before and during study. Similar to PUFA, these alternative treatments could also manage inattentive symptoms. Therefore, subjects will be withdrawn from the study if these supplements are taken in the past 1 month.

6. From the literature, the potential risks to the BCI may include seizure or destabilization of the individual's mood state. To minimize such risk, subjects with history of seizure or severe psychiatric disorder will be excluded in this study.

8.2 Concomitant Medications

To ensure that the subjects are not taking any of the above mentioned restricted drugs (see above section 9.1. item 3), all restricted medications taken within 1 month prior to screening visit and during

the entire study period will be recorded in the Concomitant Medications section of the Case Report Form (CRF).

8.3 Unblinding

Unblinding of the clinician is only allowed in cases of emergency.

9. Safety Measurements

9.1 Definitions

9.1.1 Adverse Events

The Pediatric Adverse Event Rating Scale will be used to assess adverse events.

9.1.2 Unanticipated Problems Involving Risk to Subjects or Others

Unanticipated Problems Involving Risk to Subjects or Others (UPIRTSO) events refers to problems, in general, to include any incident, experience, or outcome (including adverse events) that meets ALL of the following criteria:

1. Unexpected
   In terms of nature, severity or frequency is not consistent with –
   a. the research procedures that are described in the protocol-related documents, such as the DSRB approved research protocol and informed consent document; or
   b. the characteristics of the subject population being studied

2. Related (including possibly related) to participation in the research
   Possibly related means there is a reasonable possibility that the incident, experience, or outcome may have been caused by the procedures involved in the research.
   The following conditions might help assess causality:
   a. the event has a reasonable temporal relationship to the intervention,
   b. the event could not have been produced by the underlying disease states,
   c. the event could not have been due to other non-study interventions,
   d. the event follows a known pattern of response to the interventions, or
   e. the event disappears with cessation of intervention.

An event need not be serious to qualify as reportable.

9.1.3 Serious Adverse Events

A serious adverse event (experience) or reaction is any untoward medical occurrence that at any dose:

- results in death,
- is life-threatening,

- requires inpatient hospitalisation or prolongation of existing hospitalisation,
- results in persistent or significant disability/incapacity, or
- is a congenital anomaly/birth defect.

9.2 Collection, Recording and Reporting

9.2.1 AE

AEs will be collected from the time the informed consent and assent forms have been signed until the end of the study. This will include AEs spontaneously reported by the subject and / or observed by the parents and clinicians.

9.2.2 UPIRTSO

1. Urgent Reporting:
All problems involving local deaths, whether related or not, should be reported immediately – within 24 hours after first knowledge by the Investigator.

2. Expedited Reporting:
All other problems must be reported as soon as possible but not later than 7 calendar days after first knowledge by the Investigator.

9.3 Safety Monitoring Plan

The investigator is responsible for monitoring the safety of study participants who have entered this study and to take appropriate action concerning any event that seems unusual. Subjects will be followed up if:

- Subject has at least one SAE or subject withdrawn from the study as a result of an AE, until the event has been resolved, subsided, stabilized, disappeared, the event is otherwise explained, or the subject is lost to follow up
- Or, in the case of other non-serious AEs, until they complete the study or they are lost to follow up

9.4 Complaint Handling

Parents/legal guardians of the subject will be provided with the contact numbers of the PI and the DSRB secretariat. Complaints about the conduct of the study will be reported directly to the PI for immediate and proper actions.

9.5 Criteria for Stopping the Research

Subjects are not expected to experience any problems when participating in these training games. There is no invasive procedure involved. The research however may be suspended if any part of the protocol is breached or if there is any violation of standard research ethics.

Parents can undergo a review with the study psychiatrist to consider dropping out of the study if:
1. ADHD-RS raw score increases by 25% as assessed by the clinician and/or
2. CGI scores are between 5 and 7 inclusive.

10. Data Analysis

10.1 Data Quality Assurance

To ensure data quality entered data will be systematically checked by the data management staff and validated against rules which are built into the database system. Any discrepancies will be channeled to relevant parties for resolutions. All discrepancies will be resolved prior to database lock and data analysis. Quality control audits of all key safety and efficacy data in the database will be made before database lock.

11. Sample Size and Statistical Method

11.1 Determination of Sample size

Based on a moderate effect size of 0.5 on ADHD Rating Scale inattentive score improvement rated by clinicians, power of 80% and type I error of 5%, a sample size of approximately 80 subjects per group is necessary. This includes an allowance for up to 20% losses to follow-up.

11.2 Statistical Analysis Plans

11.2.1 General considerations

All statistical analyses will be carried out on an intention-to-treat (ITT) basis unless otherwise stated. An ITT set is defined as all randomized subjects. The treatment group of subjects in the ITT set is the planned treatment group, i.e., according to the randomization list planned prior to the study commencement. All tests of hypotheses will be two-tailed. Statistical significance testing will be performed at the 0.05 level. All statistical analyses will be performed using SAS Version 9.3 or higher. Statistical analysis and programming support will be provided by the Singapore Clinical Research Institute (SCRI).

11.2.2 Demographic and baseline characteristics

Demographic data and baseline characteristics will be summarized by descriptive statistics and presented by randomisation group. Continuous variables will be summarized using mean and standard deviation. Categorical variables will be summarized using the number of observations and percentages. Eligibility criteria deviation and subject's accountability will be tabulated and reported in CONSORT (Consolidated Standards of Reporting Trials) flow chart.

11.2.3 Analysis of primary and secondary endpoints

Change in the inattentive score on the ADHD Rating Scale as rated by clinicians at week 8 will be compared between the intervention group (post-BCI) and control groups (pre-BCI) using the two sample t test. Mean difference of the changes between the two groups and its associated 95% confidence interval will be reported. Comparison of change scores for the continuous secondary outcomes at week 8 between the two groups will also be conducted by using the two sample t test or Mann-Whitney U test, where appropriate. Chi squared test or Fisher's exact test, where appropriate, will be used for categorical secondary outcomes.

Booster effect will be analyzed by first pooling paired data before and after the boosters in the two groups. Change in efficacy endpoints as rated by clinicians and parents at week 20 from week 1 in the intervention arm and changes at week 28 from week 9 in the waitlist control group. These pooled changes before and after the boosters will be summarized; and comparisons of efficacy endpoints pre and post boosters will be made in the pooled data by using the paired t test or Wilcoxon signed rank test, where appropriate. The above will also be conducted at week 24 from week 1 in the intervention arm and week 32 from week 9 in the waitlist control to assess the effect the sustainability of the effect of the BCI system.

The longitudinal ADHD ratings, and other clinician's scales will be explored by the generalized estimating equations approach, both assessing the time trend and the difference in time trend between groups.

Frequency and percentage of the adverse events highlighted in the side effect checklist will be summarized for the pooled sample since the BCI program is administered to all subjects for all those that receive at least one BCI session.

12. Ethical Considerations

The Principal Investigator has the sole responsibility in ensuing that this clinical trial will be conducted in accordance with the applicable laws and regulations and in accordance with the good clinical practices guidelines. Only the DSRB's approved version of the protocol, assent form and informed consent form will be used throughout the study. Any amendments or revisions to the study procedure and/or consent and assent forms will only be implemented after approval from the DSRB has been granted.

Participants will be paid $40 for each interview done at baseline (week 1), and weeks 8, 20 and 24 for intervention group and weeks 1, 8, 16, 28 and 32 for control group . Participants will be paid $10 per visit for all other scheduled trial visits.

12.1 Informed Consent by the Parent and Informed Assent by the Child

A written informed consent by the parent/legal representative and a written informed assent form by the child will be obtained first before any subject is enrolled into this study. In obtaining and documenting informed consent and assent form, the Investigator or Co-investigator should adhere to the principles as stipulated in Singapore Guideline Good Clinical Practice (SGGCP).

Informed consent from parents and assent from children will be taken during the first clinic visit (pre-screening visit) at the clinic premises of the CGC. Only the Site Principal Investigator and those delegated by him to take informed consent and assent can conduct the consent process. The parents/legal guardians and subjects will be given time to consider their participation in the study. Verbal and written information given to the subject and parents/legal guardians shall be in simple terms and in a language understood by them. A copy of the signed and dated written informed consent and assent form will be provided to parent and subject/child.

To protect the privacy interests of the research participants, where “privacy interests” refer to interests of individuals to be left alone, free from intrusion and comfort with the proposed clinic settings, the child shall be accompanied by the parent/legal guardian during every visit to the clinic and all interventions will be performed in designated laboratory rooms within the clinic to ensure privacy.

12.2 Confidentiality of Data and Patient Records

All electronic data will be password-protected and essential trial documents will be kept securely with locks.

Access to research study databases will be restricted. Only study staff and staff from Singapore Clinical Research Institute (SCRI) who are involved in conducting external monitoring visits will have access to study data files. Besides, NHG Domain Specific Review Board (DSRB) and the Ministry of Health will also have access to study database for audit purposes. All patient identifiers will be delinked, and the subjects will be assigned a number instead.

Atentiv Incorporated (USA), who holds the patent and license to the Brainpal™ technology, will have access to the data generated from the study. In addition, the study data may be provided to third parties, including commercial entities for commercial use. However, no personal information that could be used to identify you or your child will be disclosed to any of these parties and will remain confidential.

No part of the study procedures will be recorded on audiotape, film/video, or other electronic medium.

13. Publication Policy

Publication of the results by the Investigator(s) is subject to mutual agreement between the Investigators and Institution. Manuscripts and abstracts must be sent to the institutional representative prior to submission.

14. Retention of Trial Documents

After completion of the study SCRI will return all study documents to the Principal Investigator for archiving according to established Institutional policy and GCP guidelines.

15. Insurance

The study will be conducted at the Institute of Mental Health and as such will be covered by the NHG Clinical Trials Compensation Insurance. The intervention involved in this study is non-invasive and it is not expected to result in any form of injury.

16. References

1. American Academy of Child and Adolescent Psychiatry. Practice Parameters for the Assessment and Treatment of Children, Adolescents, and Adults with ADHD 1997. Updated 2007.

2. Barkley RA, Fischer M, Edelbrock CS, Smallish L. The adolescent outcome of hyperactive children diagnosed by research criteria, I: an 8-year prospective follow-up study. J Am Acad Child Adolesc Psychiatry. 1990; 29: 546-557.

3. Barkley RA, Fischer M et al. The persistence of attention deficit/hyperactivity disorder into young adulthood as a function of reporting source and definition of disorder. J Abnorm Psychol 2002; 111(2):279-89.

4. Biederman J, Mick E, Faraone SV, et al. Influence of gender on attention deficit hyperactivity disorder in children referred to a psychiatric clinic. Am J Psychiatry. 2002; 159: 36-42.

5. Eric Taylor et al: European clinical guidelines for hyperkinetic disorder – first upgrade. European Child & Adolescent Psychiatry, Vol. 13, Supplement 1 (2004)

6. MTA Cooperative Group. National Institute of Mental Health Multimodal Treatment Study of ADHD follow-up: changes in effectiveness and growth after the end of treatment. Pediatrics 2004; 113; 762-769.

7. Molina BS et al. The MTA at 8 years: prospective follow-up of children treated for combined-type ADHD in a multisite study. J Am Acad Child Adolesc Psychiatry. 2009 May; 48(5):461-2.

8. Hirshberg LM, Chiu S, Frazier JA. Emerging brain-based interventions for children and adolescents: overview and clinical perspective. Child Adolesc Psychiatric Clin N Am 2005; 14; 1–19.

9. Friel, Patrick N. EEG Biofeedback in the Treatment of Attention Deficit/Hyperactivity Disorder. Alternative Medicine Review 2007; 12; 2; 146-51.

10. Monastra VJ. Electroencephalographic biofeedback (neurotherapy) as a treatment for attention deficit hyperactivity disorder: rationale and empirical foundation. Child Adolesc Psychiatr Clin N Am. 2005 Jan; 14(1):55-82.

11. Strehl U, Leins U, Goth G, Klinger C, Hinterberger T, Birbaumer N. Self-regulation of slow cortical

potentials: a new treatment for children with attention-deficit/hyperactivity disorder. Pediatrics. 2006 Nov; 118(5):e1530-40.

17. Appendices

17.1 List of Abbreviations

ADHD	Attention Deficit Hyperactive Disorder
ADHD-RS	Attention Deficit Hyperactive Disorder – Rating Scale
AE	Adverse Event
BCI	Brain-Computer Interface
CBCL	Child Behaviour Checklist
C-DISC	Computerized Diagnostic Interview Schedule for Children
C-GAS	Clinical Global Assessment Scale
CGC	Child Guidance Clinic
CGI-I	Clinical Global Impression Improvement Scale
CGI-S	Clinical Global Impression Severity Scale
CONSORT	Consolidated Standards of Reporting Trials
CPT	Continuous Performance Test
CRA	Clinical Research Associate
CRF	Case Report Form
DSM-IV TR	Diagnostic & Statistical Manual of Mental Disorders, 4th Edition Text Revision
DSRB	Domain Specific Review Board
EEG	Electroencephalogram
IQ	Intelligence Quotient
KBIT-2	Kaufman Brief Intelligence Test, 2nd Edition
LSP	Learning Support Program
MCQ	Multiple Choice Question

NHG	National Healthcare Group
PAERS	Pediatric Adverse Event Rating Scale
REACH	Reaching Everyone for Active Citizenry at Home
SCRI	Singapore Clinical Research Institute
SGGCP	Singapore Guideline for Good Clinical Practice
TRF	Teacher Report Form
UPIRTSO	Unanticipated Problems Involving Risk To Subjects or Others
