# Supplementary material for: A randomized controlled trial of a brain-computer interface based attention training program for ADHD
Source: PLoS One. 2019 May 21;14(5):e0216225. doi: 10.1371/journal.pone.0216225 (PMC6528992; doi:10.1371/journal.pone.0216225)
Supplement: S1 File — (PDF) [file pone.0216225.s003.pdf]

S1 File. BCI Neurofeedback System

Colour Stroop Task (Calibration)

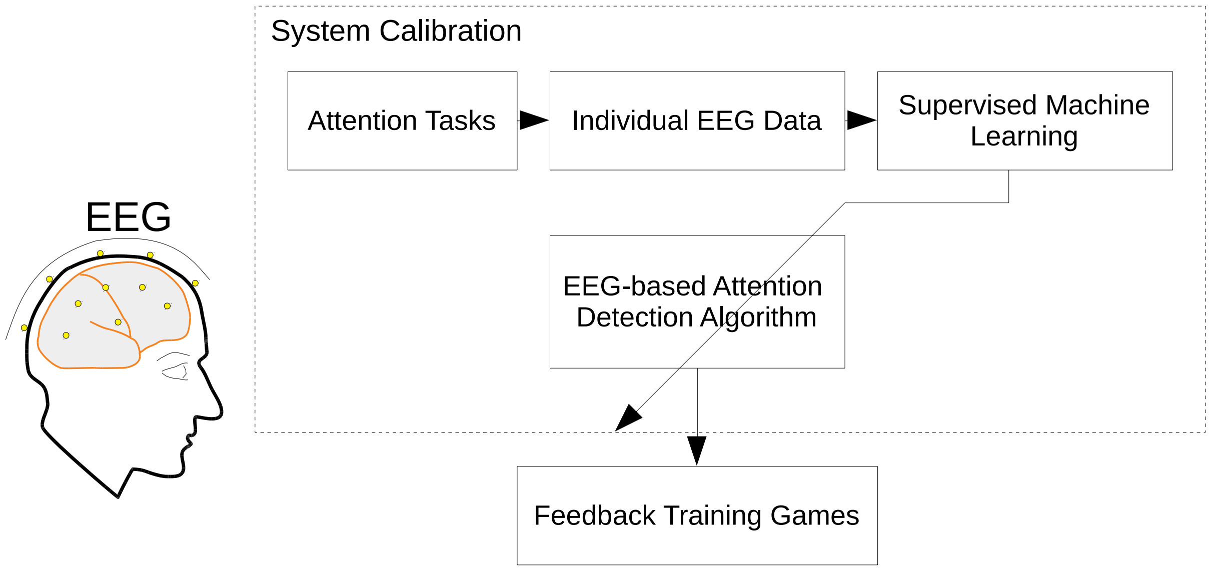

System Diagram of the BCI Neurofeedback Attention Training Method

A simplified system diagram is depicted above. The system undergoes a calibration process for each new user before the user can receive the neurofeedback training. The purpose of the calibration is to adapt an attention detection algorithm to the user's EEG characteristics so as to estimate the attention activity underlying the EEG waveforms.

Particularly, the system first guides the user through a sequence of attention tasks consisting of incongruent Stroop tests and inattentiveness tasks (e.g. idle). The incongruent colour-word Stroop tests are meant to capture the brain signal of activated executive function. Hence, we set a mandatory minimum correctness rate for the Stroop test responses at 80%, failing that would lead to a redo of the calibration session.

Concurrent EEG waveforms are recorded during the tasks, and stored into an individual EEG data set. In this study, we used a single channel EEG headband device that measured the bipolar EEG across the two frontal channels Fp1 and Fp2. The system first runs a threshold-based mechanism to detect and reject EEG samples corrupted by eye-blinking and other significant artefacts. It then runs a filter-bank mechanism to decompose the EEG waveform into 8 components corresponding to 8 frequency ranges from 4 Hz to 30 Hz, covering theta, alpha, and beta rhythms.

Hence, a 2-second shifting window is applied to the EEG frequency components to extract time-windowed EEG from the duration of each Stroop test or each inattentiveness task. The average band power of the 2-second windowed EEG at each channel-frequency component is then calculated. In our case of single-channel EEG, there are eight band power numbers to represent the spectrum feature of the EEG in each 2-second window.

The spectrum feature (an eight-element vector) samples are then categorised into attentiveness samples if the EEG segment is from a Stroop task, or into inattentiveness samples if the EEG segment is from a non-invasiveness task. We use a mutual information based criterion[1] to select the optimum set of features that discriminate attentiveness samples from inattentiveness samples. We then train a linear support vector machine (SVM) to transform the selected features into attention score, where a negative value indicates likelihood of inattentiveness and a positive value of attentiveness.

Therefore, the system learns from the data set the optimum mechanism for an online processing algorithm, including the spectrum feature set and the SVM, to predict the attentional state from EEG.

BCI Neurofeedback Attention Training

This BCI-based training is related to neurofeedback. Neurofeedback represents a promising non-invasive intervention to normalize or compensate for neuropsychological/behavioral dysfunctions associated with ADHD[2]. However, there is lack of conclusive evidence for neurofeedback's effectiveness. At the same time, the efficacy varies considerably between-patient and between-study[3], including fMRI neurofeedback studies[4]. A more recent paper[5] further proposes that, in order to rigorously estimate the earning effects of neurofeedback, the variables influencing these effects should first be identified, and recent brain-computer-interface results could be highly relevant.

Conventional neurofeedback usually selects a particular EEG rhythmic power from a particular channel for the feedback signal. By contrast, our proposed training method takes a more human-factor-centred standpoint, and adapts the training protocol to each user's neurophysiological profiles. Particularly, our method uses a brain-computer-interface technology to identify each individual user's distinguishing EEG patterns associated with different attention tasks, by using machine learning techniques on a calibration data set from the user performing a sequence of attention tasks. As a result, the new feedback signal may comprise an optimum combination of multiple rhythmic activities from one or multiple channels, and may capture each user's attention-neurophysiological profile more accurately.

REFERENCES

1. K.K. Ang ZYC, H.H. Zhang and C.T. Guan, editor Filter bank common spatial pattern (FBCSP) in Brain-computer interface. IEEE International Joint Conference on Neural Networks; 2008; United States.
2. Albert J, Sánchez-Carmona, A.J., Fernández-Jaén, A. & López-Martín, S. Neurofeedback for ADHD: A critical review and suggested future directions. Current Developmental Disorders Reports. 2017;4(3):86-93. doi: DOI 10.1007/s40474-017-0117-y.
3. Alkoby O, Abu-Rmileh, A., Shriki, O., & Todder, D. Can we predict who will respond to neurofeedback? A review of the inefficacy problem and existing predictors for successful EEG neurofeedback learning. Neuroscience. 2018;378:155-64.
4. Thibault RT, MacPherson, A., Lifshitz, M., Roth, R.R. & Raz, A. Neurofeedback with fMRI: A critical systematic review. NeuroImage. 2018;172:786-807.
5. Jeunet C, Lotte,F., Batail, J., Philip, P., Franchi, J.M. Using recent BCI literature to deepen our understanding of clinical neurofeedback: A short review. Neuroscience. 2018;378:225-33.
