# Supplementary material for: A randomized controlled trial of a brain-computer interface based attention training program for ADHD
Source: PLoS One. 2019 May 21;14(5):e0216225. doi: 10.1371/journal.pone.0216225 (PMC6528992; doi:10.1371/journal.pone.0216225)
Supplement: S2 File — (PDF) [file pone.0216225.s004.pdf]

S2 File. Secondary Efficacy Analysis: Clinician rated CGAS, CGI-S and CGI-I

The clinician completed the Children's Global Assessment Scale (CGAS) and Clinical Global Impression Scale that measures illness severity (CGI-S) and global improvement (CGI-I) at each assessment visit. The Children's Global Assessment Scale (CGAS) provide a global measure of the child's level of functioning, based on a 100-point score, with a higher score representing higher level of functioning. The CGI is rated on a 7-point scale, with the CGI-S using a range of responses from 1 (normal) through to 7 (amongst the most severely ill patients). The CGI-I scores range from 1 (very much improved) through to 7 (very much worse), with the mid-point score of 4 representing 'no change'.

To address the secondary efficacy of BCI treatment effect, two-sample independent t-test was used to compare the mean change in CGAS. For CGI-S, the Mann-Whitney U-test was used to test for differences in the distribution of change scores. In CGI-I, we dichotomized the CGI-I outcome into into "improved" (CGI-I assessments of "*much improved*" or "*very much improved*") and "not improved" (CGI-I assessments of "*minimally improved*", "*no change*", "*minimally worse*", "*much worse*" or "*very much worse*") and then conducted a test of equality of proportions using the Fisher exact test. Additionally, the risk ratio was computed and a 95% confidence interval constructed. We performed these analyses on a modified intent-to-treat population that is all randomized patients who had non-missing Week 8 assessments, irrespective of eligibility status or compliance with protocol, and analysing them under the group they were randomized to.

On the CGAS which were rated by the blinded clinicians, both groups showed significant improvement from pre-BCI states at the end of the entire trial. The intervention group showed a mean improvement of 4.3 (SD 5.87) over their baseline score of 56.6 (SD 4.29), while the control group showed a mean improvement of 4.6 (SD 5.81) over their

baseline score of 57.5 (SD 4.78). Pooling both groups, the mean improvement in functioning was 4.5 points (95% CI 3.5 to 5.4, p<0.0001).

Based on the CGI-S, the median change scores indicate no change in severity in both groups, although based on the range of change scores, more spread in the direction of improvement is seen in the BCI compared to the Waitlist Control group. Pooling both groups, there is sufficient evidence to reject the null hypothesis of no difference in the distribution of pre-BCI and post BCI scores (at end of trial) (p<0.0001). Post BCI scores tend to be distributed at the low (less severity) rather than high (greater severity) end of the CGI-S.

Based on the CGI-I, clinicians assessed that 24.7% and 31.9% improved in the child's clinical symptoms in the BCI-intervention and Waitlist control groups respectively. Pooling both groups, 28.3% (95% CI 21.6 to 36.1) improved four weeks after the end of the booster training.

Improvements found from CGAS, CGI-S and CGI-I was in line with the study's secondary efficacy analyses of clinician-rated ADHD-RS and parent-rated CBCL.
