# Supplementary figures and images for: A randomized controlled trial of a brain-computer interface based attention training program for ADHD
Source: PLoS One. 2019 May 21;14(5):e0216225. doi: 10.1371/journal.pone.0216225 (PMC6528992; doi:10.1371/journal.pone.0216225)

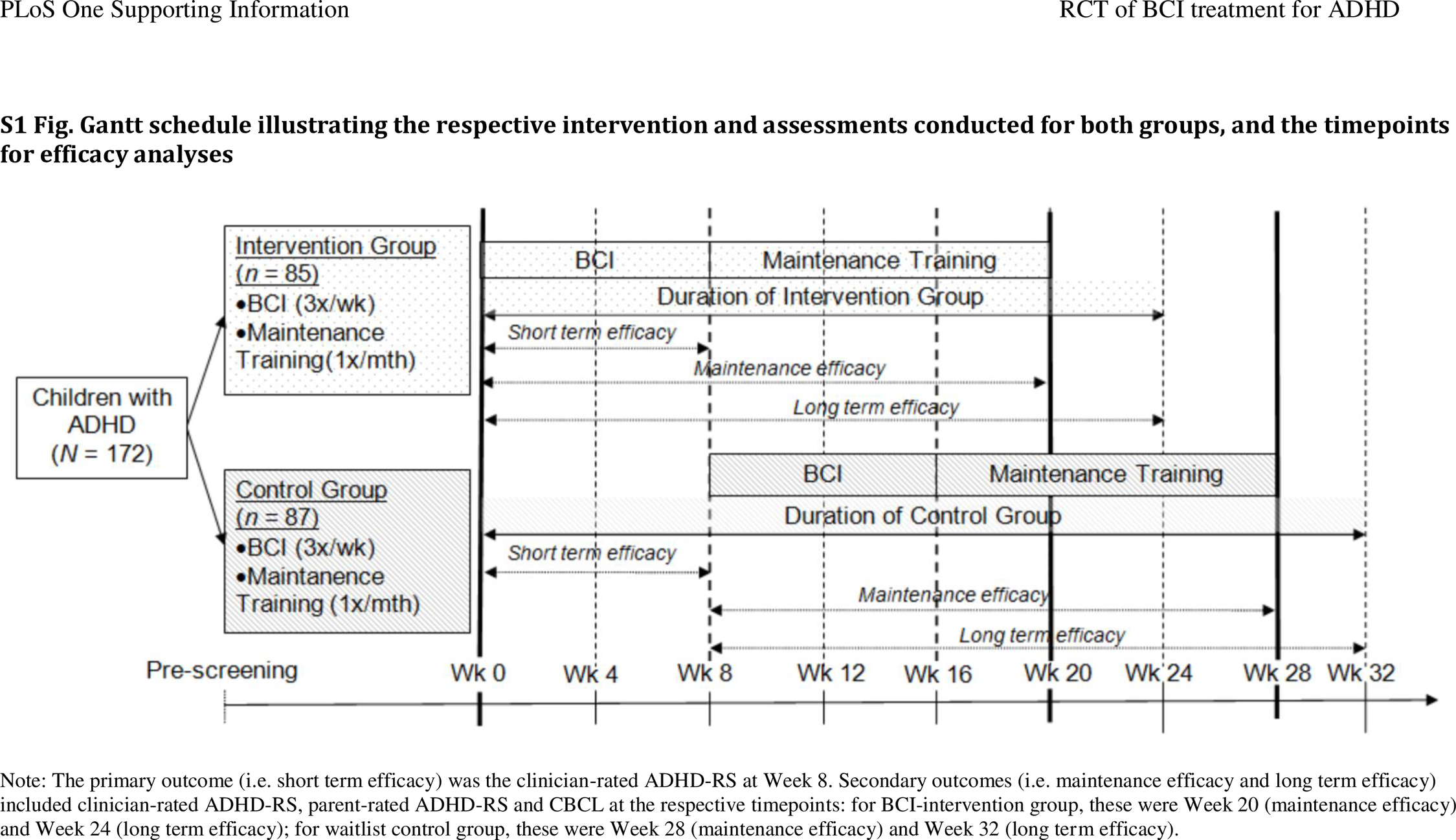

Supplement: S1 Fig — The primary outcome (i.e. short term efficacy) was the clinician-rated ADHD-RS at Week 8. Secondary outcomes (i.e. maintenance efficacy and short efficacy) included clinician-rated ADHD-RS, parent-rated ADHD-RS and CBCL at the respective timepoints: for BCI-intervention group, these were Week 20 (maintenance efficacy) and Week 24 (long term efficacy); for waitlist control group, these were Week 28 (maintenance efficacy) and Week 32 (long term efficacy). (TIF) [file pone.0216225.s005.tif]
