# Supplementary material for: A randomized controlled trial of a brain-computer interface based attention training program for ADHD
Source: PLoS One. 2019 May 21;14(5):e0216225. doi: 10.1371/journal.pone.0216225 (PMC6528992; doi:10.1371/journal.pone.0216225)
Supplement: S1 Table — (PDF) [file pone.0216225.s007.pdf]

S1 Table. Secondary Efficacy Analysis: Clinician-rated CGAS, CGI-S and CGI-I

Clinician-rated CGAS	Mean	SD	Mean	SD	Mean Difference (95% CI); p-value
Week 0	56.6	4.29	55.7	4.48	
Short-term efficacy (post 8-wk training): Δ* at Week 8	2.8	4.75	1.8	4.92	1.03 (-2.6 to 0.5); p=0.1817 [a]
End of Maintenance training: Δ* at wk 20/28	3.2	6.03	3.4	5.19	3.3[b] (2.4 to 4.2); p<0.0001[c]
Long-term (end of trial): Δ* at Week 24/32	4.3	5.87	4.6	5.81	4.5[b] (3.5 to 5.4); p<0.0001[c]
**Clinician rated CGI-S**	**Median**	**Min, Max**	**Median**	**Min, Max**	**Median change score (Min, Max); p-value**
Week 0	4.0	0.0, 5.0	4.0	3.0, 5.0	
Short-term efficacy (post 8-wk training): Δ* at Week 8	0.0	-5.0, 1.0	0.0	-1.0, 2.0	0.2026[d]
End of Maintenance training: Δ* at Week 20/28	0.0	-2.0, 5.0	0.0	-2.0, 1.0	0.0 (-2.0, 5.0); p<0.0001[e]
Long-term (end of trial): Δ at Week 24/32	0.0	-2.0, 5.0	0.0	-2.0, 1.0	0.0 (-2.0, 5.0); p<0.0001[e]
**Clinician rated CGI-I**	**%**	**95% CI**	**%**	**95% CI**	**% (95% CI)**
Short-term efficacy (post 8-wk training): % improved at Week 8***	11.5	5.4 to 20.8	3.8	0.8 to 10.6	0.0773[f]; Risk ratio 3.0 (95% CI 0.86 to 10.94)
End of Maintenance training: % improved at Week 20/28***	21.6	12.9 to 32.7	16.7	8.9 to 27.3	19.2 (13.1 to 26.5)[g]
Long-term (end of trial): % improved at Week 24/32***	24.7	15.3 to 36.1	31.9	21.4 to 44.0	28.3 (21.6 to 36.1)[g]

[*] Δ=change score; Δ > 0 indicates improvement, Δ=0 no change and Δ < 0 deterioration; in Waitlist control group only, Δ is calculated from Week 8 maintenance training and long-term sustainability (end of trial).

** Δ=change score ; Δ > indicates increase, Δ = no change and Δ < 0 decrease in functioning; in Waitlist control group only, Δ is calculated from Week 8 for maintenance training and long-term sustainability (end of trial).

*** Improvement is defined as CGI-I ratings of "very much improved (CGI-I =1)" or "much improved (CGI-I=2)", all others defined as no improvement (CGI-I ≥ 3)

[a] Mean difference, independent two-sample t-test

[b] Mean of change scores from pooled BCI-Intervention and Waitlist control groups

[c] Paired t-test result to test null hypothesis that mean change score is zero

[d] Mann-Whitney U-test for a difference in distributions of CGI-S change scores between BCI-intervention and Waitlist control groups;

[e] Wilcoxon signed-rank test p-value to test null hypothesis that pre-BCI and post-BCI scores are sampled from the same population

[f] Fisher's exact test p-value; risk ratio is

[g] percentage of participants in the pooled BCI-Intervention and Waitlist control who were assessed by clinician as either "very much improved" (CGI-I = 1) or "much improved" (CGI-I=2) and 95% confidence interval
