# Supplementary material for: A randomized controlled trial of a brain-computer interface based attention training program for ADHD
Source: PLoS One. 2019 May 21;14(5):e0216225. doi: 10.1371/journal.pone.0216225 (PMC6528992; doi:10.1371/journal.pone.0216225)
Supplement: S2 Table — (PDF) [file pone.0216225.s008.pdf]

S2 Table. Summary of adverse events for all subjects who underwent at least one BCI session

	Number of participants	Number of adverse events
At least one adverse event	11	18
Dizziness	4	4
Headache	6	12
Motor restlessness	1	1
Trouble paying attention/concentrating	1	1
Severity, n		
Mild	8	14
Moderate	3	4
Function impaired, n		
No	11	18
Intervention-related, n		
Yes	8	13
No	3	5
Outcome, n		
Recovered/Resolved	11	18
